# 6-Bromo quinazoline derivatives as cytotoxic agents: design, synthesis, molecular docking and MD simulation

**DOI:** 10.1186/s13065-024-01230-2

**Published:** 2024-07-04

**Authors:** Leila Emami, Maryam Hassani, Pegah Mardaneh, Fateme Zare, Maryam saeedi, Mina Emami, Soghra Khabnadideh, Sara Sadeghian

**Affiliations:** 1 Pharmaceutical Sciences Research Center, Shiraz University of Medical Sciences, Shiraz, Iran; 2 Department of Medicinal Chemistry, Faculty of Pharmacy, Shiraz University of Medical Sciences, Shiraz, Iran; 3 Medicinal and Natural Products Chemistry Research Center, Shiraz University of Medical Sciences, Shiraz, Iran

**Keywords:** Cancer, Quinazoline-4(3*H*)-one, MTT, Docking, MD, DFT

## Abstract

Based on unselectively, several side effects and drug resistance of available anticancer agents, the development and research for novel anticancer agents is necessary. In this study, a new series of quinazoline-4(3*H*)-one derivatives having a thiol group at position 2 of the quinazoline ring **(8a-8 h)** were designed and synthesized as potential anticancer agents. The Chemical structures of all compounds were characterized by ^1^H-NMR, ^13^C-NMR, and Mass spectroscopy. The antiproliferative activity of all derivatives were determined against two cancer cell lines (MCF-7 and SW480) and one normal cell lines (MRC-5) by the MTT method. Cisplatin, Erlotinib and Doxorubicin were used as positive controls. The results of in vitro screening showed that **8a** with an aliphatic linker to SH group was the most potent compound with IC_50_ values of 15.85 ± 3.32 and 17.85 ± 0.92 µM against MCF-7 and SW480 cell lines, respectively. **8a** indicated significantly better potency compared to Erlotinib in the MCF-7 cell line. The cytotoxic results obtained from testing compound **8a** on the normal cell line, revealing an IC_50_ value of 84.20 ± 1.72 µM, provide compelling evidence of its selectivity in distinguishing between tumorigenic and non-tumorigenic cell lines. Structure–activity relationship indicated that the variation in the anticancer activities of quinazoline-4(3*H*)-one derivatives was affected by different substitutions on the SH position. Molecular docking and MD simulation were carried out for consideration of the binding affinity of compounds against EGFR and EGFR-mutated. The binding energy of compounds **8a** and **8c** were calculated at -6.7 and − 5.3 kcal.mol^− 1^, respectively. Compounds **8a** and **8c** were found to establish hydrogen bonds and some other important interactions with key residue. The DFT analysis was also performed at the B3LYP/6–31 + G(d, p) level for compounds **8a**, **8c** and Erlotinib. Compound **8a** was thermodynamically more stable than **8c**. Also, the calculated theoretical and experimental data for the IR spectrum were in agreement. The obtained results delineated that the **8a** can be considered an appropriate pharmacophore to develop as an anti-proliferative agent.

**Supplementary Information:**

The online version contains supplementary material available at 10.1186/s13065-024-01230-2.

## Introduction

Cancer remains one of the major health problems around the world which is associated with a high mortality rate and is the second cause of death after cardiovascular diseases [1, 2]. Cancer is a group of diseases characterized by uncontrolled growth and proliferation of abnormal cells that have the potential to spread to other tissues throughout the body [3, 4]. Unfortunately, there has been a steady increase in the annual incidence rate of cancer globally. In 2020, 19.3 million new cases of cancer and approximately 10.0 million deaths were reported. Furthermore, it is estimated that the number of new cancer cases might reach 28.4 million in 2040 [5, 6]. This has raised an urgent need for the development of preventive or therapeutic strategies. Different approaches including immunotherapy, hormone therapy, surgery, radiotherapy, and chemotherapy have been used to treat cancer, among which chemotherapy is the most common and effective approach [7, 8]. Chemotherapy agents kill cancer cells by disrupting the cell cycle by one or multiple pathways, either by directly inhibiting DNA or RNA synthesis or by affecting key proteins involved in the cell cycle [7, 9].

A wide range of chemotherapeutic drugs alone or in combination with other drugs are mainly used for the treatment of various cancers. Despite the wide range of anticancer drugs that are available in clinics, several significant challenges including lack of selectivity, induction of multi-drug resistance and toxic side effects hindered the efficiency of chemotherapy [3, 10]. Therefore, the discovery and development of novel anticancer agents to address the challenges above of chemotherapy are urgently needed. On this basis, substantial efforts have been made by various researchers to develop novel anticancer agents by incorporating various active functionalities into chemical scaffolds [11–13].

Quinazoline is one of the excellent scaffolds used in the design and synthesis of new biologically active compounds with diverse biological activities [14–16]. Indeed, quinazoline derivatives have shown various biological activities such as anticancer, antimicrobial, antihypertensive, antihyperlipidemic, anti-inflammatory, and anticonvulsant activities [16–19].

**Fig. 1:**
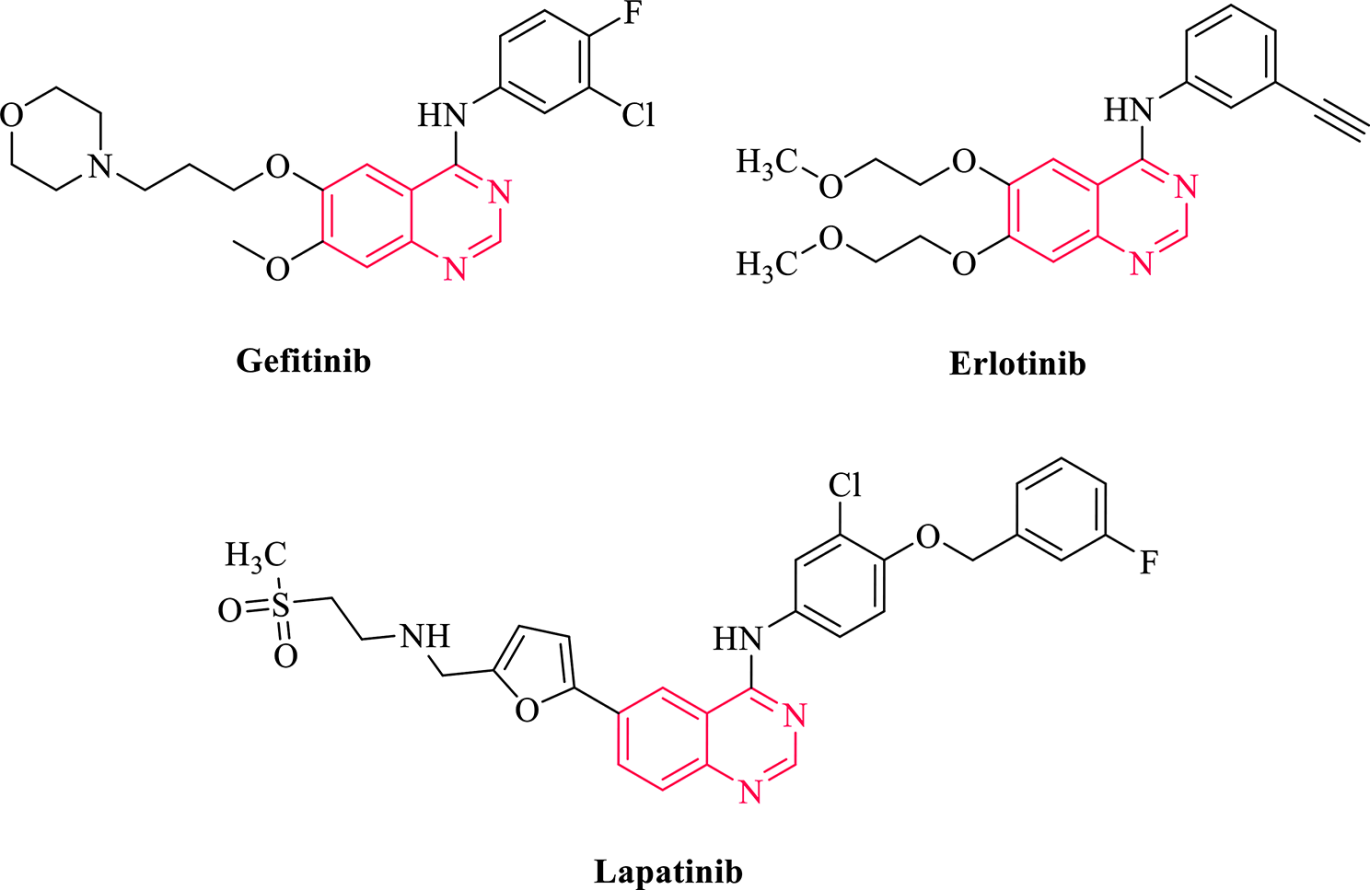
FDA-approved quinazoline-based drugs

In recent years, several quinazoline based drugs, like Gefitinib, Erlotinib, and Lapatinib have been approved by the FDA as anticancer drugs (Fig. 1) [10]. Among various quinazoline derivatives 2-thioxo-3-substituted quinazolinones and their S-methyl thioether counterparts, as well as 6-substituted quinazolinone derivatives showed promising anticancer activity [16, 20–22]. According to the literature, 3-position substituted quinazoline derivatives can exert their cytotoxic activities through inhibition of EGFR as an important target in cancer [23–25].

**Fig. 2:**
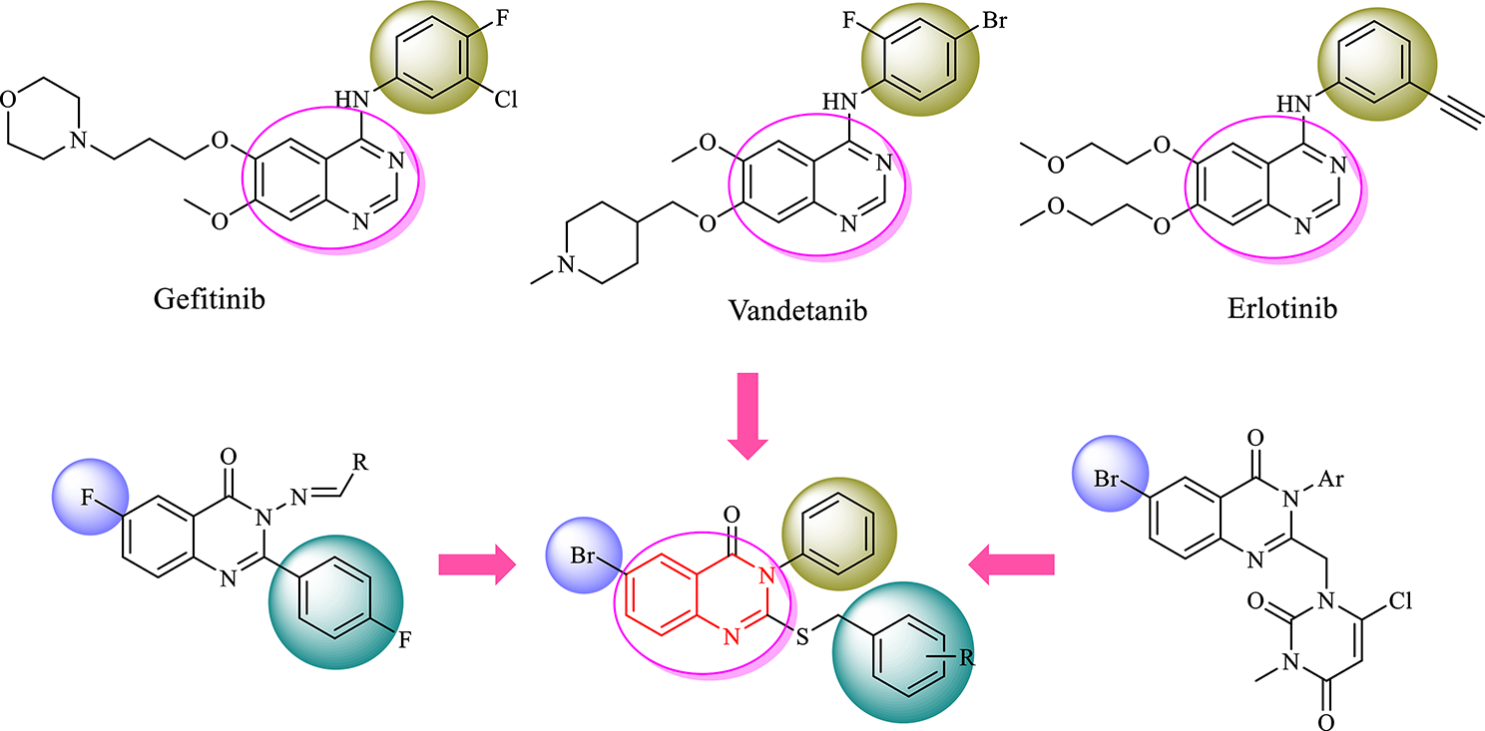
The design strategy of the target compounds

Quinazoline and quinazolinone scaffolds as active frameworks in the design of anticancer agents have been the subject of numerous studies [26, 27]. Currently, a number of quinazoline-based drugs such as Gefitinib, Erlotinib, Afatinib, etc. have been approved by FDA for the treatment of cancer. Studies have also shown that the presence of halogen atom at the 6-position of quinazoline ring can improve anticancer effects [28, 29]. Therefore, in the present study, a rational template with quinazoline-4-one scaffold as pharmacophoric group was designed which includes a thio-benzyl moiety at 2-position of quinazoline ring and a phenyl moiety at 3-position of the quinazoline ring to improve the electronic and hydrophobic interactions with the active site of enzyme (Fig. 2).

We also synthesized and evaluated some quinazoline derivatives as anticancer agents in our previous works [29–32]. In continuation of our work on the synthesis of quinazoline derivatives, herein, several new quinazoline-4(3*H*)-one derivative (**8a-8 h**) were synthesized and evaluated for their in vitro cytotoxicity against MCF-7 and SW480 cell lines. Cisplatin, Erlotinib and Doxorubicin were used as positive controls. Additionally, a molecular docking study and MD simulation were performed to predict the possible binding mode of these compounds in the active site of EGFR as the possible receptor. DFT analysis was also carried out to provide useful information about the reactivity of the molecule in the various reactions.

## Results and discussion

### Chemistry

**Fig. 3:**
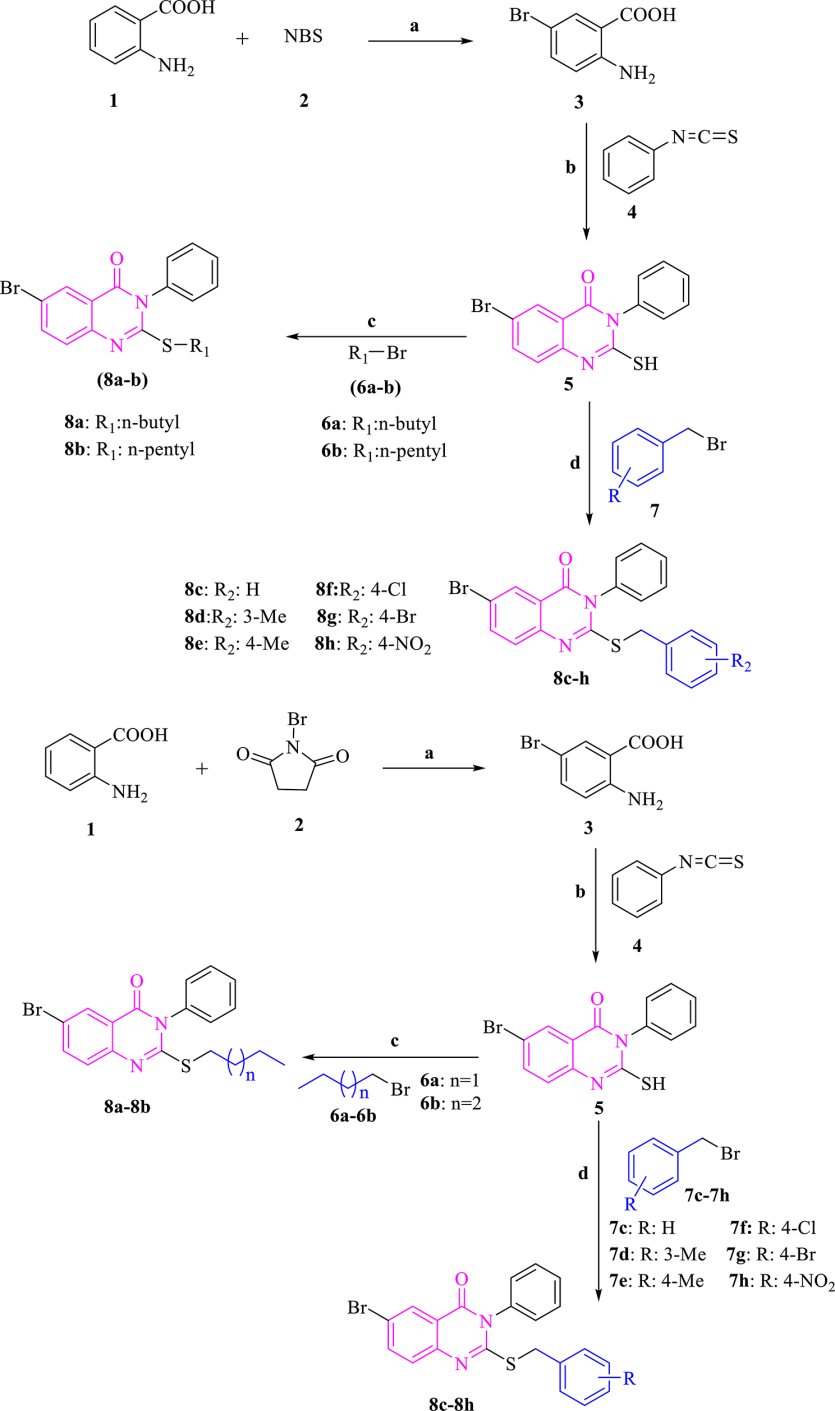
Synthesis of compounds **8a-8 h**. Reagents and conditions: (**a**) CH_3_CN, r.t., 2 h; (**b**) EtOH, Et_3_N, reflux, 20 h; (**c**) DMF, K_2_CO_3_, reflux, 24 h; (**d**) DMF, K_2_CO_3_, reflux, 24 h

The reaction of anthranilic acid **(1)** and N-bromosuccinimide (NBS) (**2)** in acetonitrile afforded compound **3** which was then reacted with phenyl isothiocyanate (**4)** in ethanol to obtain the key intermediate 6-bromo-2-mercapto-3-phenylquinazolin-4(3*H*)-one (**5)**. The reaction of intermediate **5** with various alkyl halides or substituted benzyl bromides in DMF and the presence of K_2_CO_3_ resulted in the corresponding quinazoline-4-one derivatives (**8a-8 h)** in high yields (Fig. 3). The structures of the target compounds were identified using ^1^H-NMR, ^13^C-NMR, and Mass spectroscopy. In the ^1^H-NMR spectra of the synthesized compounds, the two benzylic hydrogens of compounds **8c**-**8 h** were observed as a singlet signal at δ = 4.4 ppm with the integration of two protons. Three protons of methyl group in compounds **8d** and **8e** appeared at δ = 2.3 ppm. In the ^13^C-NMR spectra, carbonyl peak appeared at *δ* = 160 ppm. Furthermore, the benzylic CH_2_ group of compounds **8c**-**8 h** appeared at *δ* = 37 ppm and the methyl group of compounds **8d** and **8e** appeared at *δ* = 21 ppm. In addition, in the Mass spectra of the synthesized compounds, the molecular ion is in agreement with the molecular weight of the synthesized compounds. The details of spectroscopic data are listed in the supplementary data.

### Biological activity

**Fig. 4:**
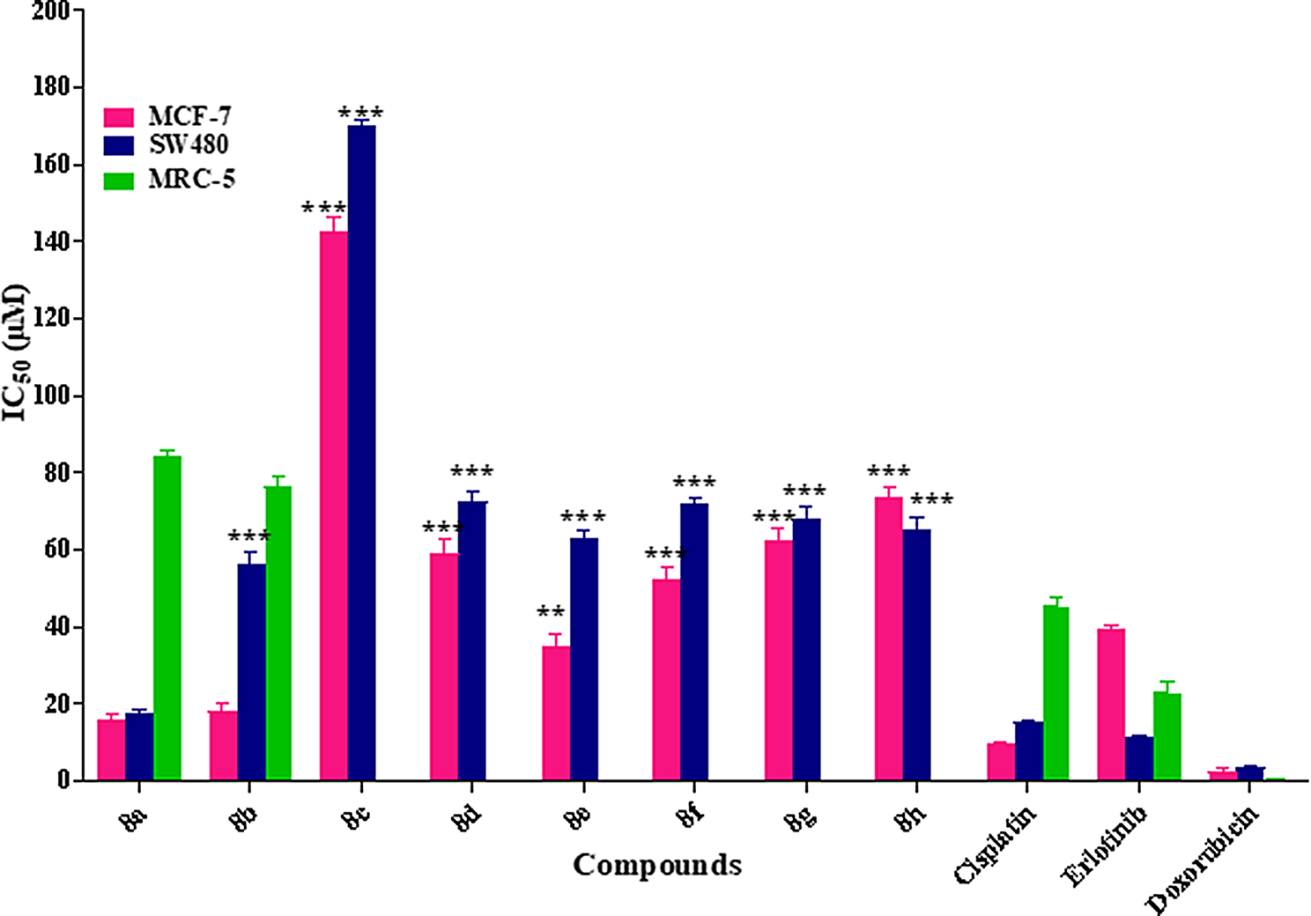
Cytotoxic activity of all the synthesized compounds against MCF-7, SW480 and MRC-5 cell lines∗, ∗∗, and ∗∗∗ indicate *p* < 0:05, *p* < 0:01, and *p* < 0:001 respectively compared to cisplatin

In vitro cytotoxic activity of the novel quinazoline-4(3*H*)-one derivatives were assayed by the MTT standard method. The results are presented in Table 1; Fig. 4. All compounds represented the less cytotoxic effect on normal cell (MRC-5) compared to MCF-7 and SW480 cell lines, that indicated appropriate selectivity between tumorigenic and non-tumorigenic cell lines. These results delineate that compound **8a** IC_50_ (15.85 ± 3.32) against MCF-7 Cell line is more potent than Erlotinib with IC_50_ (9.9 ± 0.14). One possible reason for this observation could be the better accommodation of compound 8a with alkyl ring into EGFR enzyme that is agreement with the binding energies obtained from docking experiments. To better explain the structure-activity relationship, the synthesized compounds were divided into two categories based on substitutions on SH fragments (aliphatic or aromatic substitutions). Analogs with aliphatic chain (**8a-8b**) especially with 4 carbons spacer had strong potency in MCF-7 and SW480 cell lines and also, increased linker length leading to a decrease in the activity. The second category **(8c**-**8 h)** containing aryl linker represented that compound **8e** with methyl substitution on the para position of the phenyl ring had higher potency. As dedicated in Table 1, the unsubstituted derivative **(8c)** had the least activity which revealed that the existence of substitution on the phenyl ring is necessary for antiproliferative activity.

**Table 1:**
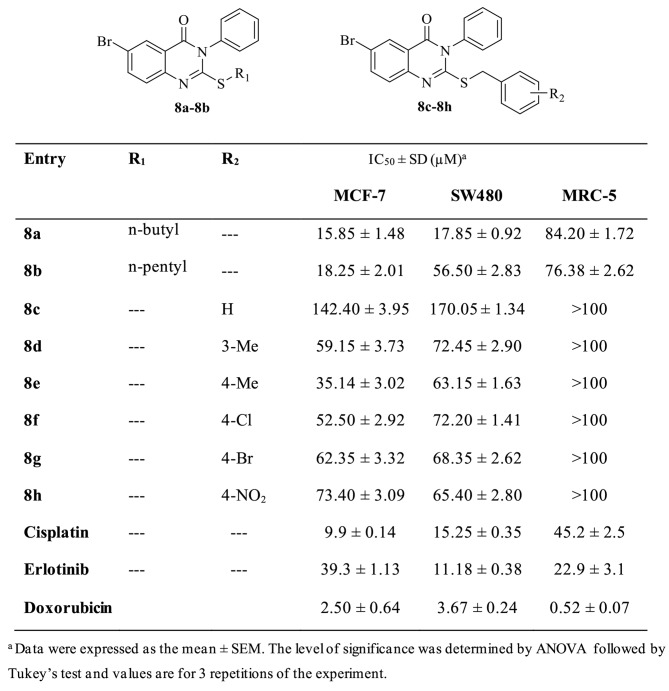
Anticancer activity (IC_50_ ± SD (µM)) of all the synthesized compounds

The presence of electron-donating groups on the phenyl ring **(8e)** resulted in an improvement in the antiproliferative activity compared to electron-withdrawing groups (**8f-8 h**). However, **(8e)** bearing methyl at para position of phenyl demonstrated better results with an IC_50_ value of 35.14 ± 6.87 µM and 63.15 ± 1.63 µM compared to the meta derivative **(8d)** with an IC_50_ value of 59.15 ± 5.73 µM and 72.45 ± 2.90 µM, against MCF-7 and SW480 cell lines, respectively. This finding indicated that placement of a methyl group at para position of the phenyl moiety increased the potency of the compound more than meta position. Evaluation of the electron withdrawing substitutions on the phenyl ring (Br, Cl, and NO_2_) represented that no significant differences were observed among them. In series of **8a**-**8 h**, the aliphatic chain-containing derivatives (**8a** and **8b**) showed cytotoxicity activity 2–8 folds more than non-substituted and compounds with aromatic chain. On the other hand, **8a** exhibited better potency vs. Cisplatin and Erlotinib as positive control. It was understood that the aromatic chain at thiol position is not tolerated. Overall, compounds, containing alkyl linker were more potent than compounds bearing aryl linker and the compounds are more effective for MCF-7 in comparison with the SW480 cell line, too.

### Molecular docking study

**Fig. 5:**
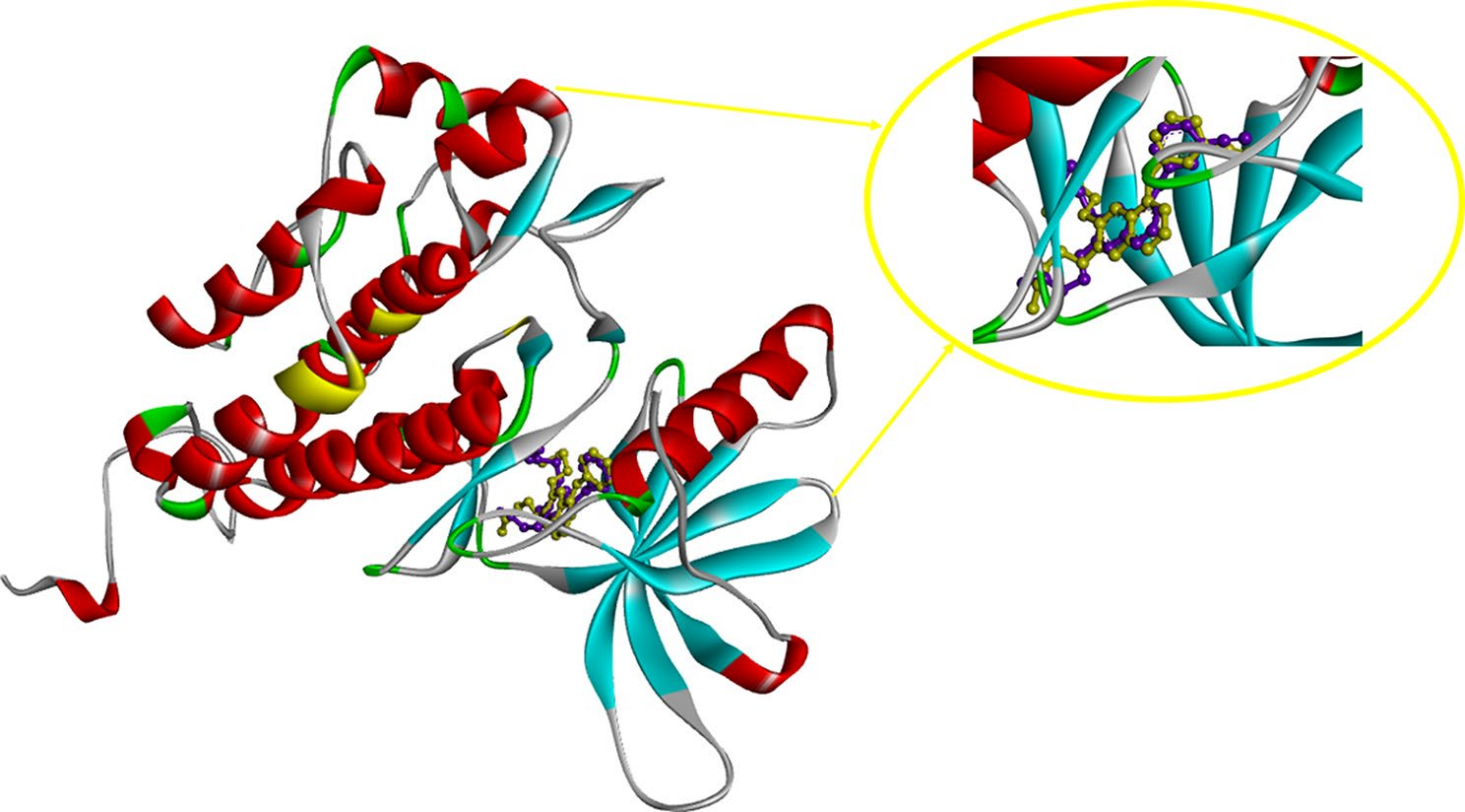
Superimposition of Erlotinib in the active site of EGFR (PDB: 1M17): yellow color indicated the redocked model and the purple color illustrated the crystal orientation

Molecular docking study was performed to understand the binding sites and interactions of the studied ligands in the active site of the EGFR target as a plausible mechanism. Redocking of the co-crystal ligand (Erlotinib) was done to evaluate the docking process and RMSD of docking was found to be 1.78. The result is presented in Fig. 5.

**Fig. 6:**
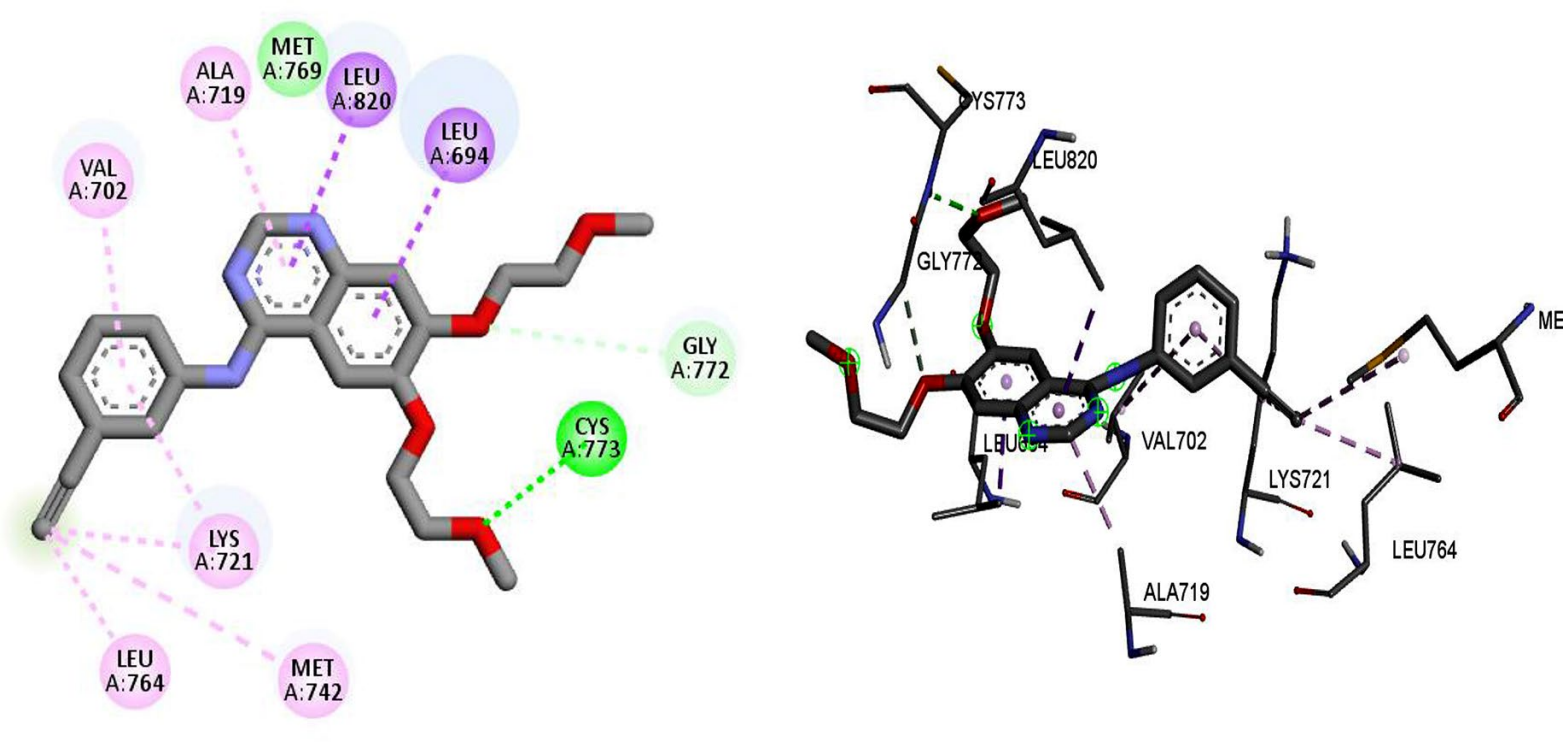
Interactions and orientation of Erlotinib in the active site of 1M17 (green: hydrogen bond, light green: van der Waals, purple: pi-pi, pink: pi-alkyl)

The types of interactions and binding poses of Erlotinib as an internal ligand in the active site of 1M17 protein are shown in Fig. 6. Hydrogen bond interactions with the residues of Cys773, and Met769 and also π-sigma interactions with Leu820 and Leu694 were observed. Some alkyl and π-alkyl interactions with Val702, Ala719, Leu764, Lys721, and Met742 were also, seen in Fig. 6.

**Fig. 7:**
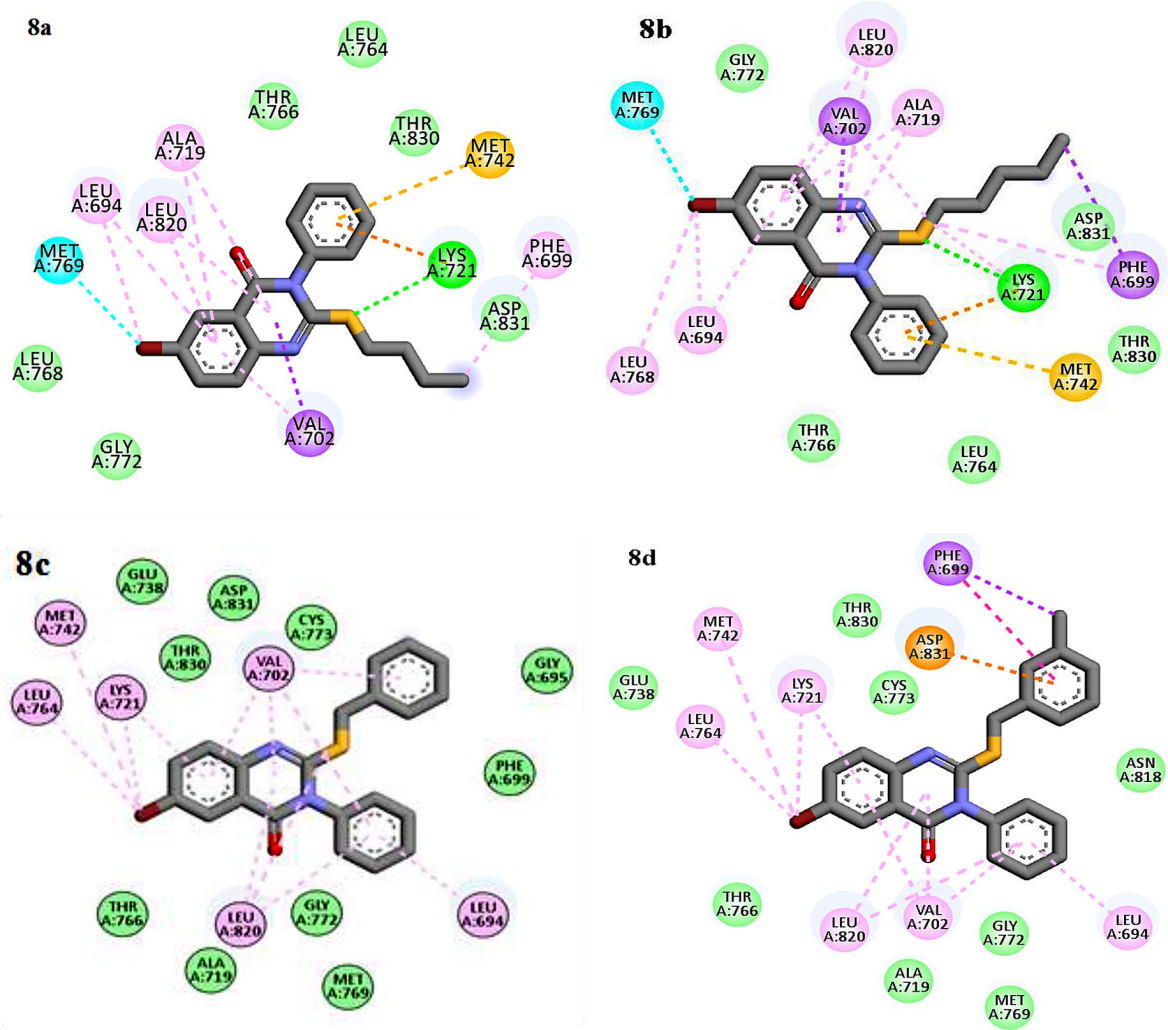
Interactions and orientation of the compounds **8a-8d** in the active site of 1M17 (green: hydrogen bond, light green: van der Waals, blue: halogen bond, orange: π-sulfur, purple: π-π, pink: π -alkyl)

**Fig. 8:**
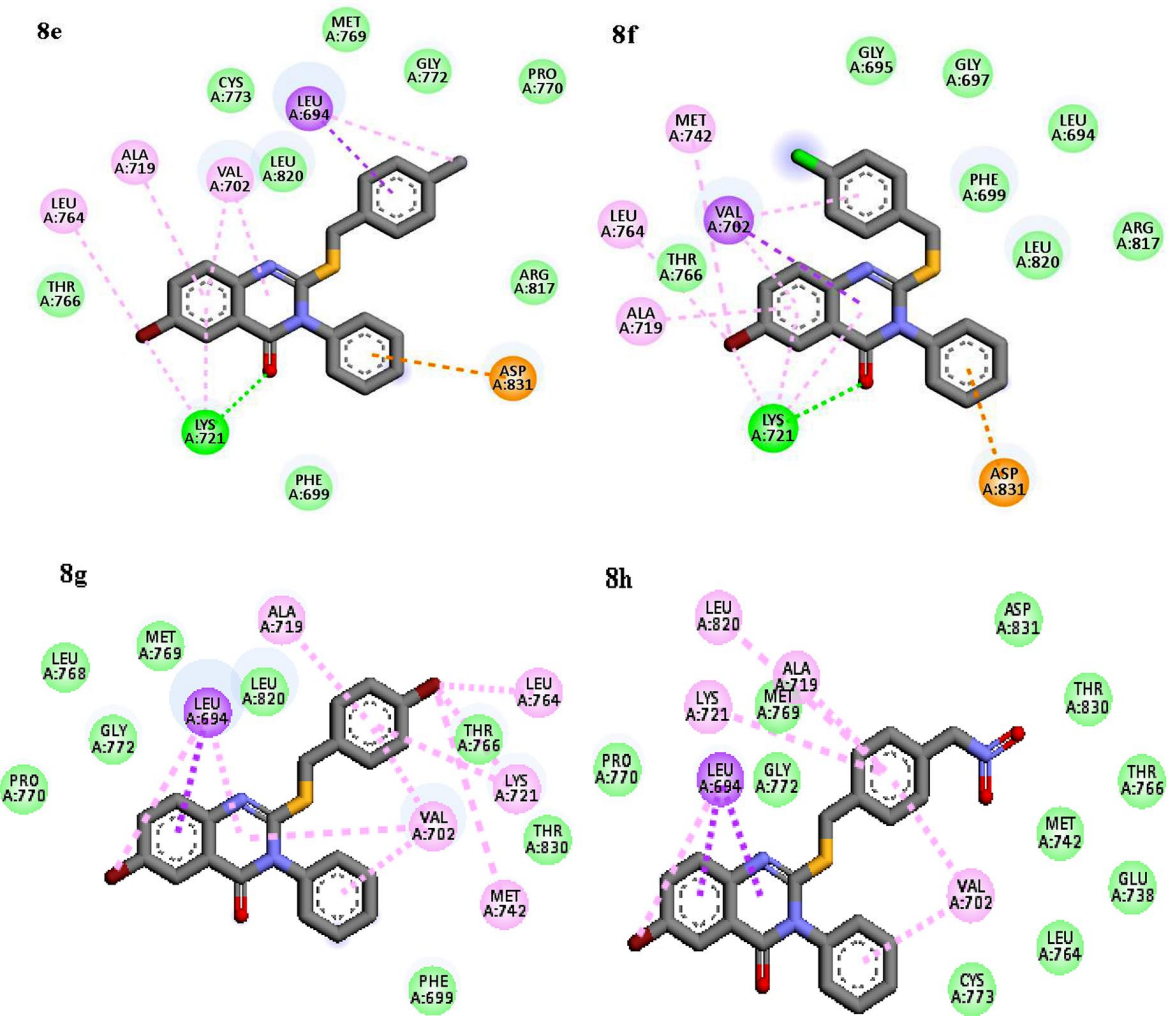
Interactions and orientation of the compounds **8e-8 h** in the active site of 1M17 (green: hydrogen bond, light green: van der Waals, purple: π- π, pink: π-alkyl)

As it is shown in, Figs. 7 and 8, based on docking results, common interactions such as π - π or π -alkyl and hydrophobic interactions with Thr 766, Thr 830, Leu 764, Phe 699, Val 702, Gly 772, Ala 719, Leu 694 and Leu 820 were seen in all compounds which had benzyl moiety at thio group (**8c**-**8 h**), showed different interaction depend on substitution on benzyl ring. Compounds **8e** and **8f**, which had methyl and chlorine substitution at para position on benzyl moiety, they interacted through similar hydrogen bond interaction between carbonyl group and Lys 721 and π-anion interaction was also, seen. On the other hand, compounds **8a** and **8b** with alkyl chain were stabilized by hydrogen interactions between the thio group of the alkyl ring and Lys 721 residue and another π-sulfur and halogen bond were observed. These common and extra strong and desirable interactions with EGFR target made the **8a** and **8b** as potential inhibitors of EGFR target among all the tested compounds. Amino acid residues Lys 721, Leu 694, Leu 820, Met 742, Cys 773 are very important for the active conformation of Erlotinib. Compounds 8a and 8b also showed desirable interactions with the same residue similar to Erlotinib. This results indicate that these two compounds are well targeted the EGFR active sites and showed more inhibitory effects than the other compounds.

**Fig. 9:**
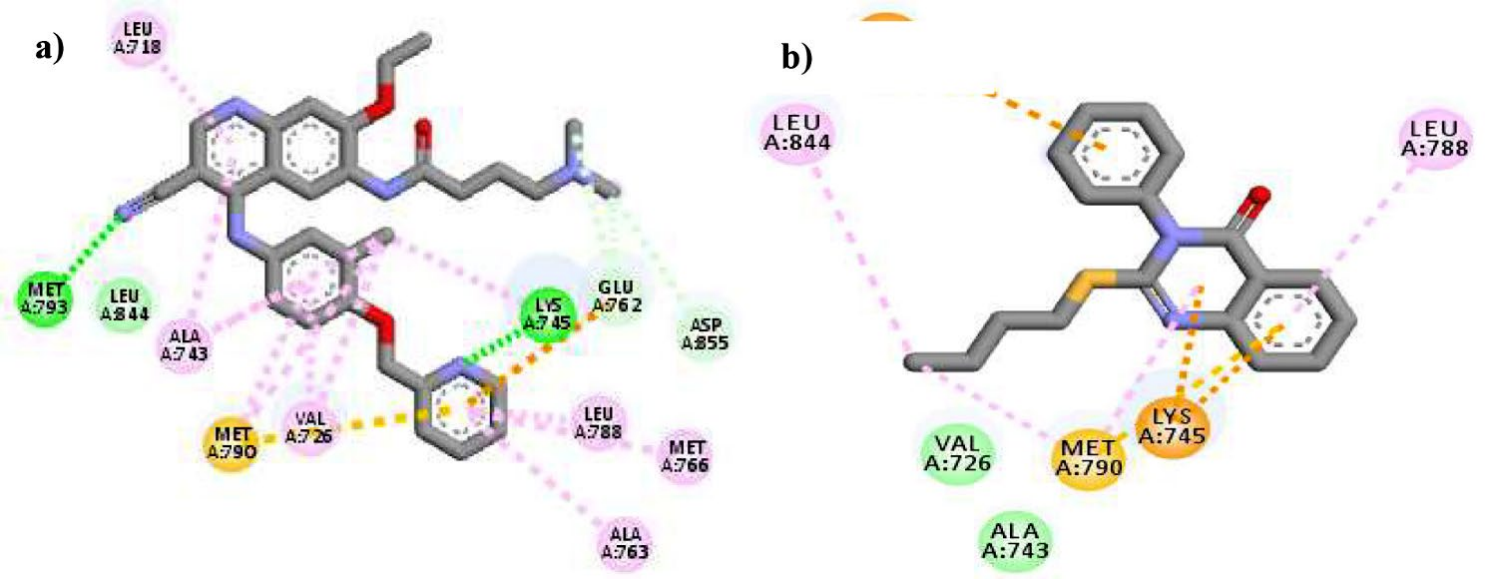
2D interactions and orientations of Naritinib (**a**) and compound **8a** (**b**) in the active site of EGFR-mutated (pdb code: 3w2q) (green: hydrogen bond, light green: van der Waals, purple: π - π, pink: pi-alkyl, orange: π -cation)

The Reports showed that mutation in EGFR increased the activity of EGFR and as a result caused a variety of cancers and resistance to EGFR inhibitor drugs, such as Erlotinib and Gefitinib. Therefore, it is necessary to consider the binding affinity of synthesized EGFR inhibitor compounds towards mutated EGFR. The interactions of the potent EGFR inhibitor, **8a**, and co-crystal ligand (Neratinib) of EGFR-mutated with the pdb code of 3w2q are displayed in Fig. 9. The total binding energy values for co-crystal ligand and **8a** were calculated at -7.1 and − 6.8 kcal.mol^-1^, respectively.

The Naritinib surrendered by residues of Leu 718, Met 793, Leu 844, Ala 743, Met 790, Val 726, Lys 745, Glu 762, Leu 788, Mt 766, Ala 763 and Asp 855. The key interactions of Naritinib were hydrogen bonding with residues of Met 793 and Lys 745 and also π contacts with Glu 762 and mutant gatekeeper residue, Met 790. Molecular docking results of the mutated-EGFR showed a well-fit binding pattern of compound **8a** with several key residues in the active site (Fig. 9). Quinazolinone ring interacted through the π interactions with Lys 745, Met 790 (the critical residues of the active site) whereas, the phenyl moiety formed π interaction with Asp 855 [33]. As can be seen, compound **8a** interacted with the Met 790, mutant gatekeeper residue as π-sulfur interaction similar to Neratinib [33].

The results indicated that the selected compound was located in the binding pocket EGFR-mutated. Consequently, the compound **8a** can inhibit both EGFR and EGFR-mutated.

**Fig. 10:**
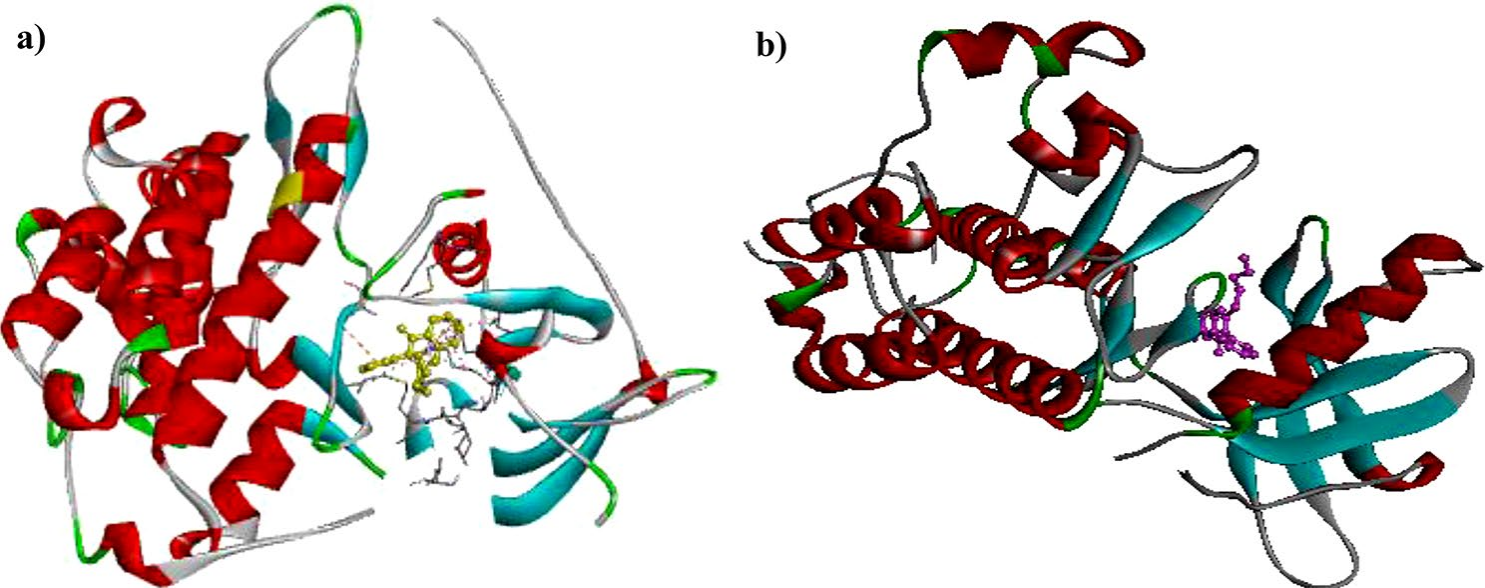
The orientations of compound **8a** in the in the active site of EGFR-mutated (pdb code: 3w2q) (**a**) and EGFR (1M17) (**b**)

No similar residues were showed in both protein so, perhaps mutant protein can change the binding pocket. The placement position of compound **8a** in the active site of EGFR-mutated (10a) and EGFR protein kinase was shown in Fig. 10.

### Molecular dynamics simulation

**Fig. 11:**
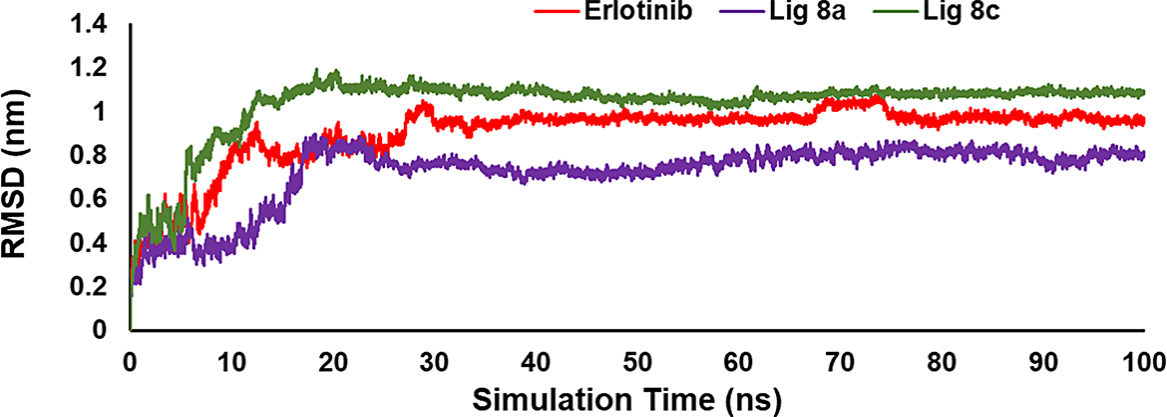
RMSD evaluation of a complex involving compound **8a** (shown in violet), **8c** (shown in green), and Erlotinib (shown in red) with EGFR kinase enzyme

Another effective approach to studying the structural modification of biomedicine molecules and intermolecular interaction patterns is molecular dynamic (MD) simulation [34]. In this study, MD simulation was conducted over a 100 ns period, exploring interactions between the atoms of compounds (**8a**, **8c**) and the native ligand Erlotinib with the EGFR receptor (1M17), as well as compound **8a** with EGFR-mutated (3W2Q). The main purpose of the simulation was to assess the conformational stability of the enzyme. Conformational stability was evaluated by calculating the Root-Mean-Square Deviation (RMSD) values, which measure the deviation of atomic positions in different frames of the simulation compared to a reference frame. The alignment was performed by comparing the backbone atoms of each frame to those of the first frame in the simulation. Based on the regular RMSD profile, compound **8a**, **8c**, and Erlotinib achieved the equilibrium phase at approximately 30, 20, and 30 ns of the simulation, respectively (Fig. 11). Additionally, compound **8a** attained the equilibrium phase at 25 ns (Fig. 15). This indicates that the ligands have settled into stable positions within the active site of the EGFR and EGFR-mutated kinase protein. The regularity of the RMSD profile suggests that the ligands remained relatively stable throughout the simulation. Furthermore, the RMSD values for both compounds **8a** and **8c**, as well as Erlotinib with 1M17, and compound **8a** with the 3W2Q protein (presumably a reference structure), demonstrate significant stability. Additionally, the RMSD values of both compound **8a** and **8c** and Erlotinib with 1M17 protein (presumably a reference structure) are significantly stable. This implies that the ligands maintain a consistent conformation and interaction with the EGFR kinase receptor throughout the simulation. The root mean mean square deviation (RMSD) of **8a** and **8c** compared to the original ligand (Erlotinib) when interacting with 1M17 was 0.7 and 1.03 Å, while for the native ligand, it was 0.9 Å throughout the simulation periods. The results indicate that ligand **8a** demonstrates better stability within the protein’s active site compared to the native ligand. In contrast, ligand **8c** exhibits less stability in comparison to Erlotinib, the native ligand [35].

**Fig. 12:**
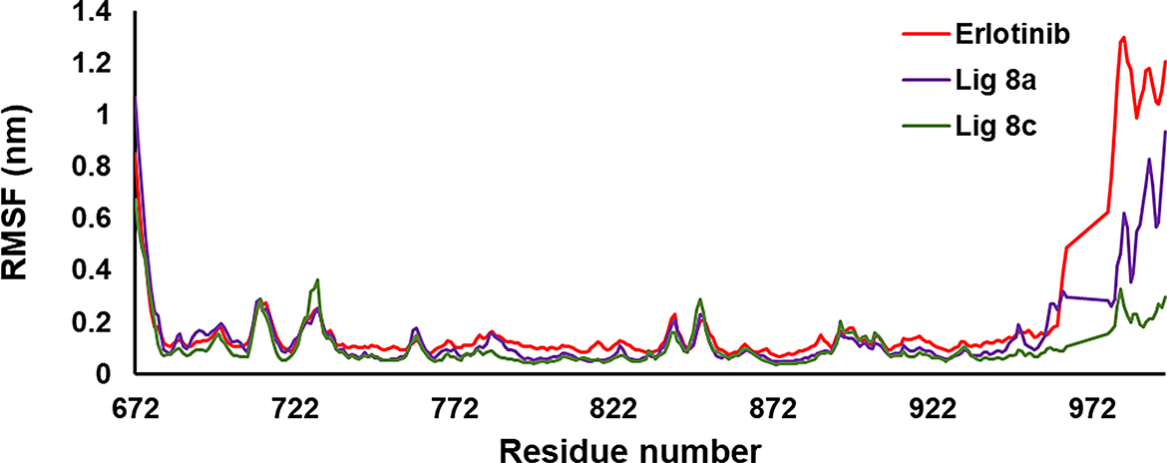
RMSF values of the complexes between **8a** (in violet), **8c** (in green) and Erlotinib (in red) in the simulation time (100 ns)

Based on the analysis of the root mean square fluctuation (RMSF) of the backbone, the flexibility of EGFR and EGFR-mutated protein was investigated to ligand binding throughout the simulation (Figs. 12 and 15). The findings indicate that complexes with **8a**, **8c**, and with the native ligand (Erlotinib) exhibited a comparable distribution of RMSF values. This suggests that the binding of either ligand does not significantly alter the overall flexibility of both the proteins. Furthermore, it was observed that no residues within the active site of the protein kinase displayed an RMSF value greater than 0.3 nm. This implied that the binding of the ligands did not induce any substantial fluctuations or changes in the flexibility of the residues within the active site. The active site remained relatively stable throughout the simulation. The RMSF values suggest that the interaction of **8a** and **8c** with the protein induces flexibility across all regions, similar to the effect observed with the native co-crystal ligand during a 100 ns simulation. The RMSF plot supports the lack of structural changes in 1M17 upon binding **8a** and **8c** compounds.

**Fig. 13:**
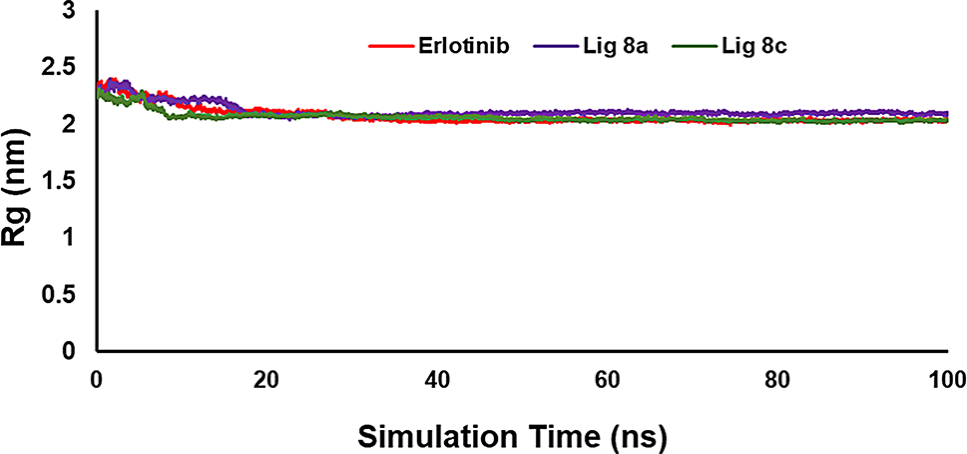
The radius of gyration (Rg) for EGFR receptors in complex with Erlotinib, **8a**, and **8c** during the simulation time

The parameter known as the radius of gyration (Rg) is used to assess the compactness changes in a ligand-protein complex [36]. The analysis revealed that the protein accumulated during the simulation time, indicating some compression of the protein. The Rg plot shows that the Erlotinib, compound **8a** and **8c** complexes have the same Rg platform, which was calculated as 2.06, 2.11, and 2.05 respectively, with the EGFR protein. The Rg values for all complexes exhibit minimal variation and maintain a low average Rg value throughout a 100 ns simulation. This consistency serves as a reliable indicator of their remarkable stability and compactness within the system. Additionally, the **8a** compound demonstrated a range of hydrogen bonds between 0 and 3 with the EGFR-mutated protein. Studying hydrogen bonding during MD simulations offers valuable insights into the interactions between molecules in ligand-enzyme systems. This analysis can greatly contribute to our comprehension of binding strength and the stability of molecular structures (Fig. 13).

**Fig. 14:**
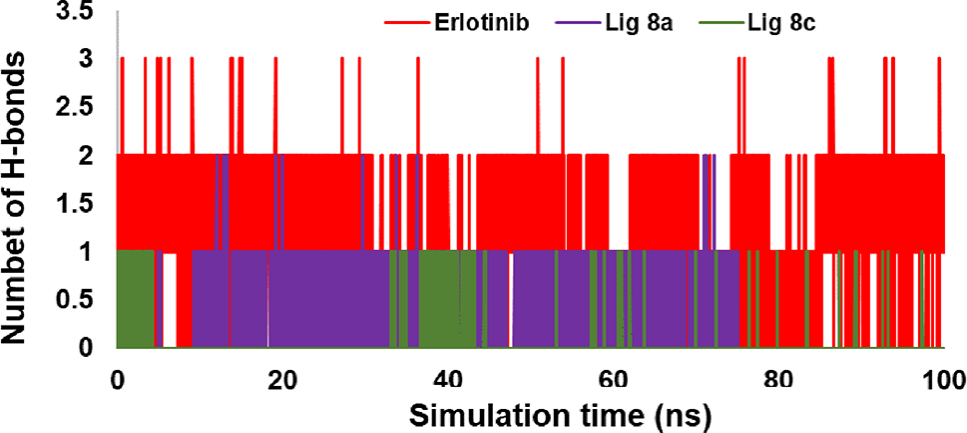
The total number of hydrogen bonds of Erlotinib, compound **8a** and **8c**

Hydrogen bonding is crucial in determining the strength of the interaction between the ligand and enzyme. To evaluate the stability of the ligand’s conformation, the number of hydrogen bonds was determined throughout the MD simulation (Fig. 14). The investigation revealed that compound **8a, 8c**, and the native ligand exhibited a range of hydrogen bonds between 0 and 2, 0 and 1, and 0 and 3, respectively. Studying hydrogen bonding during MD simulations offers valuable insights into the interactions between molecules in ligand-enzyme systems. This analysis can greatly contribute to our comprehension of binding strength and the stability of molecular structures. In general, the examination of hydrogen bond interactions between erlotinib, **8a**, and **8c** ligands and 1M17 was computed and depicted in Fig. 14. Throughout the 100 ns simulation, **8c** displayed minimal interactions with residues in the active site compared to the reference molecule (Erlotinib). On the other hand, the **8a** compound exhibited greater stability with an RMSD of 0.2 Å.

**Fig. 15:**
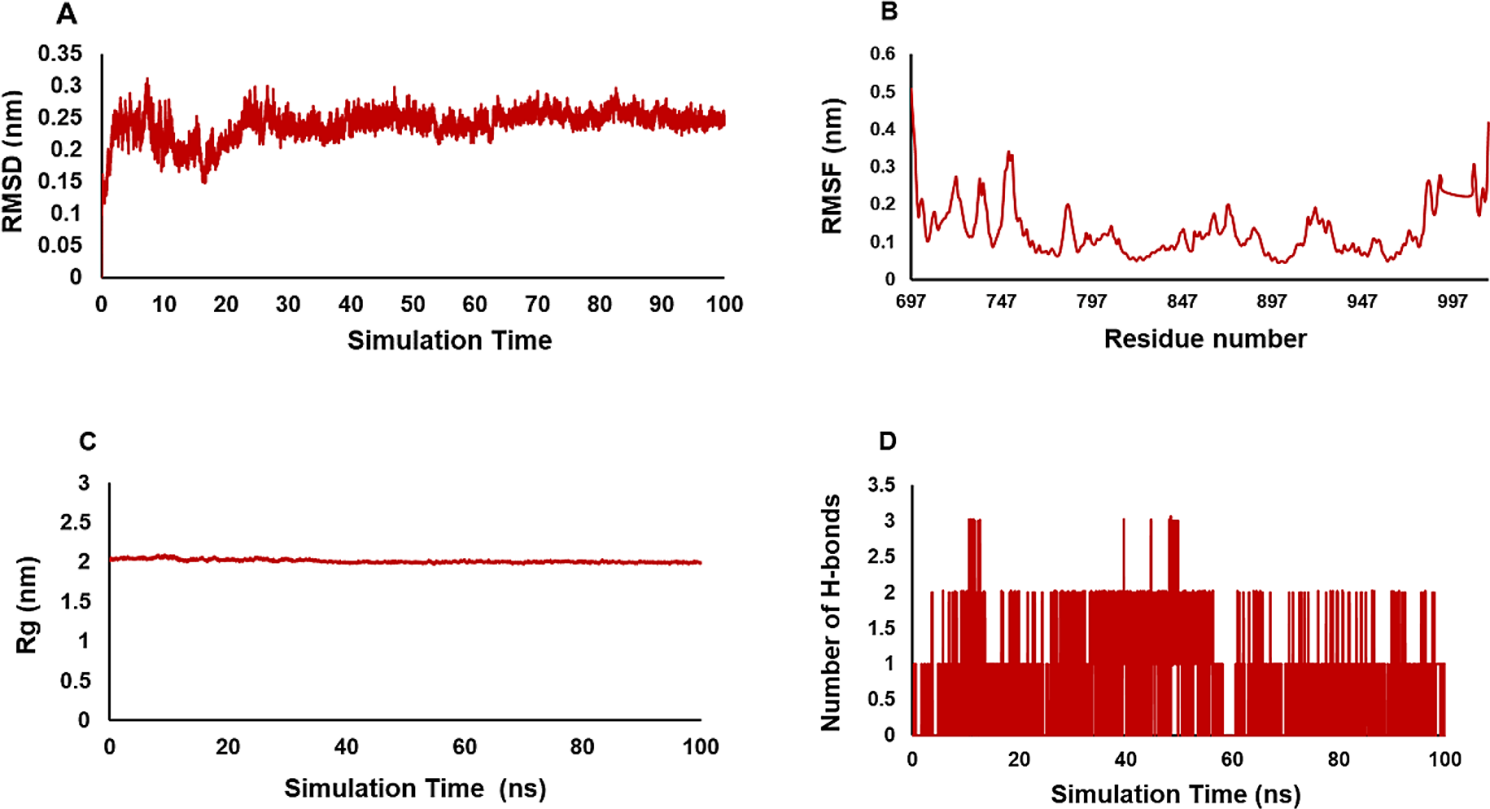
The Molecular dynamics analysis and interactions of compound **8a** with EGFR-mutated for 100 ns. (**A**) Assessment of the Root-Mean-Square Deviation (RMSD) for the complex consisting of **8a** in conjunction with the EGFR-mutated kinase (PDB: 3W2Q). (**B**) The RMSF values for the complex formed by the **8a** molecule were analyzed. (**C**) Rg plot for **8a** ligand in complex with EGFR-mutated during the simulation. (**D**) The total number of hydrogen bonds of **8a** complex

**Fig. 16:**
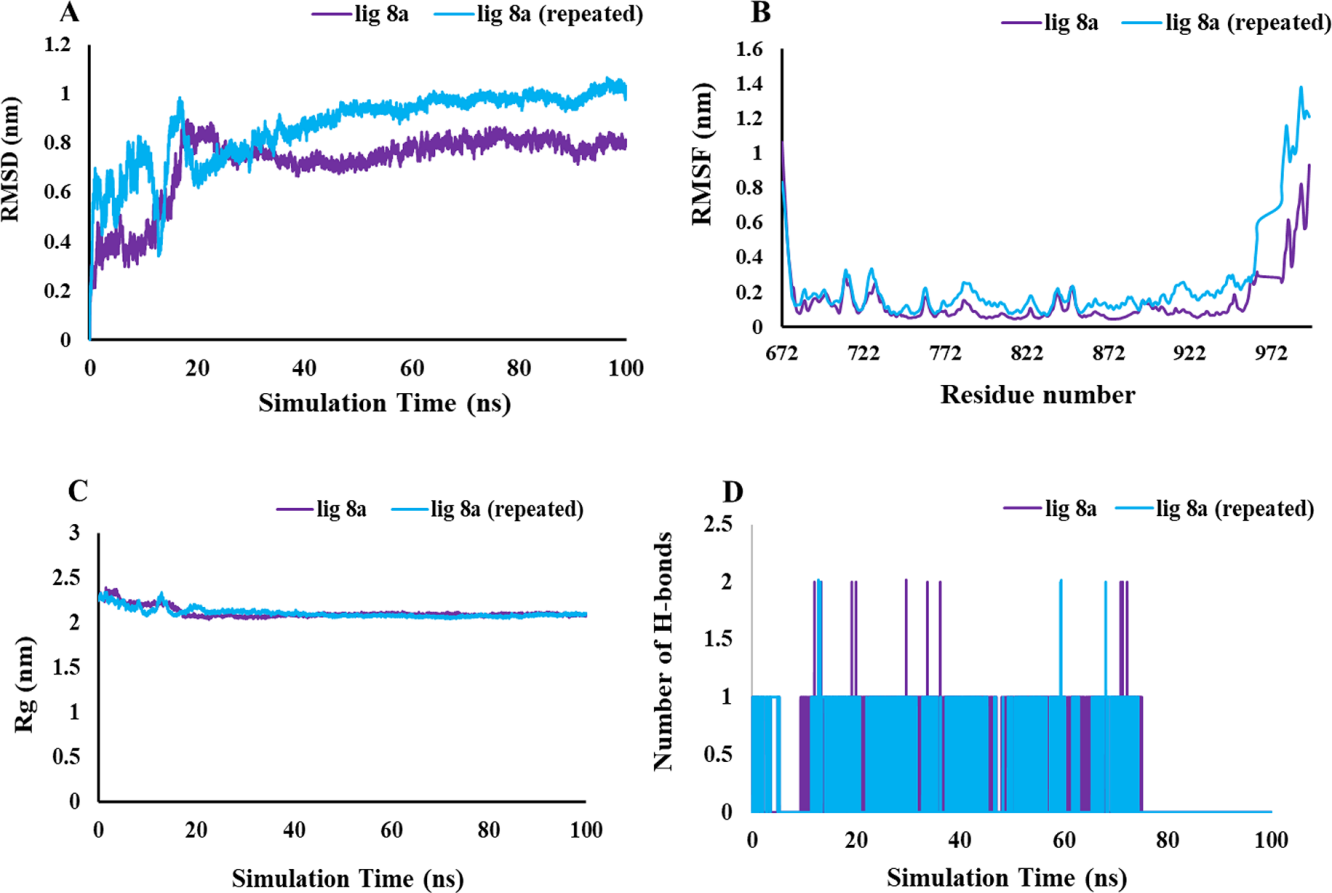
The RMSD (**A**), RMSF (**B**), Rg (**C**), and H-bonds (**D**) values are calculated for both ligand 8a and its repetitions

To evaluate the reproducibility of MD simulation, we performed two replica simulations on **8a** compound [37]. The results are justifiable and almost adaptable, suggesting that the findings have a reasonable foundation while also hinting at the potential for flexibility in their application (Fig. 16).

Overall, compound **8a** showed greater MD simulation items such RMSD, Rg, RMSF and hydrogen bond interaction compared to **8c** which confirmed higher inhibitory activity for **8a**.

### DFT analysis

**Fig. 17:**
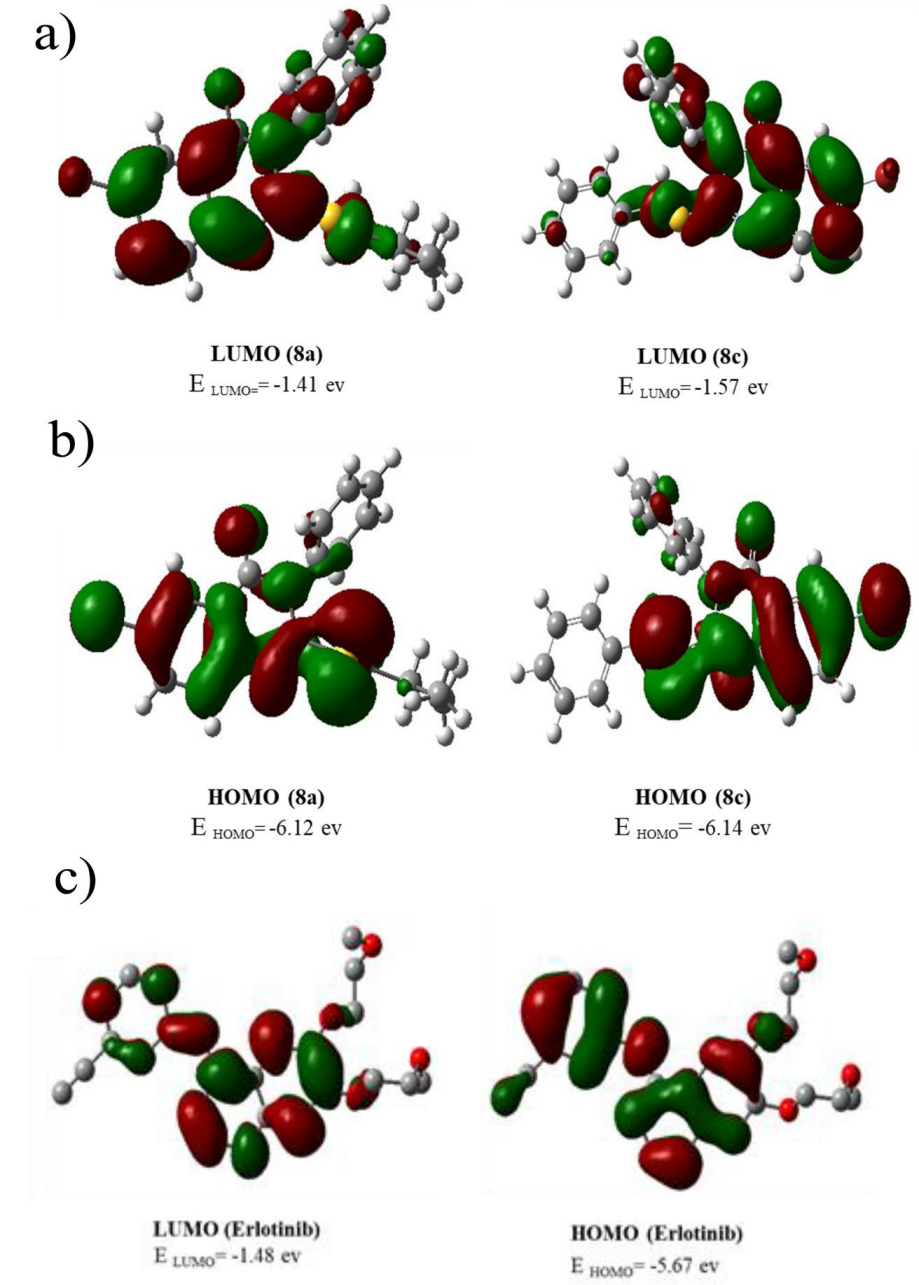
DFT calculated LUMO, HOMO, and their energies for (**a**) **8a** (left) and **8c** (right) and (**b**) Erlotinib, at the B3LYP/6–31 + G (d, p) level of theory

The measurement of the electronic parameters of a molecule can provide useful information about the behavior of the molecule in various types of reactions and also justify the results obtained from its biological activity [38]. Density functional theory (DFT) for **8a**, and **8c** as the most active and less active compounds according to the biological results and Erlotinib as reference compound was carried out with Gaussian 09 at the B3LYP/6–31 + G (d, p) level of theory. B3LYP is a hybrid density functional method that uses the Becke three-parameter exchange functional and the Lee-Yang-Parr correlation functional. It is a very popular and accurate method for quantum chemical calculations, especially for molecules with N, O, C, and H atoms. B3LYP is a GGA method, which means it includes both exact exchange and GGA corrections in addition to LDA electron-electron and electron-nuclei energy. The exact exchange term is manually adjusted to enforce the Pauli Exclusion Principle, while the GGA corrections are used to account for various effects such as dispersion, polarization, and relativistic effects. The 6–31 + G (d, p) basis set is a split-valence basis set that includes polarization functions, which are important for accurately describing the electronic structure of molecules [39–41]. The molecular orbitals, HOMO and LUMO, and their energies are shown in Fig. 16. The HOMO for both compounds **8a** and **8c** were located on two rings such as bromobenzene and quinazolinone, and sulfur atom while The LUMO distributed the charges throughout the molecule for both. As can be observed in Fig. 17, the HOMO and LUMO orbitals for Erlotinib are located in the over this molecule. The energy gap between HOMO and LUMO is a suitable measure to determine the reactivity or kinetic stability of compounds, therefore a compound with a higher energy gap is more stable and less reactive. The energy gaps between HOMO and LUMO were calculated at 4.71 and 4.57 and 4.18 ev for **8a**, **8c**, and Erlotinib respectively which indicates that compound **8a** is more stable than **8c** and Erlotinib.

**Fig. 18:**
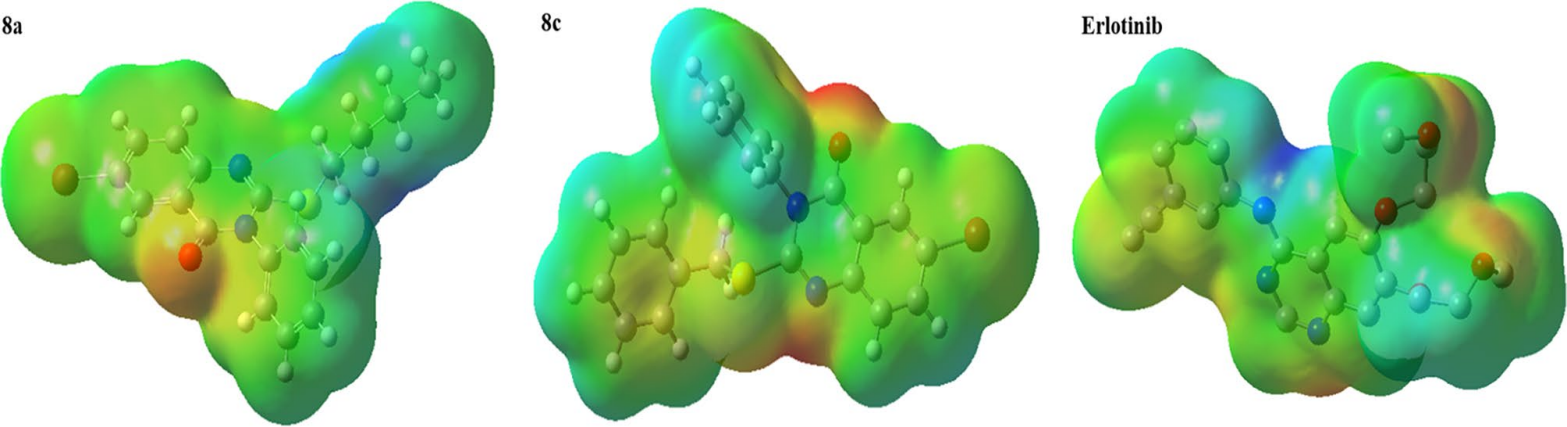
Electrostatic potential maps for **8a**, **8c** and Erlotinib at B3LYP/6–31 + G (d, p) level of theory

The electrostatic potential (ESP) map is a tool to identify the active sites of the compound for electrophilic and nucleophilic attacks. The ESP maps for compounds **8a** and **8c** are given in Fig. 18. The areas with negative electrostatic potential are indicated by red and yellow spheres, which are preferred sites for electrophilic attack, while blue and green areas have positive electrostatic potential and are suitable sites for nucleophilic attack. As can be seen, in the compounds, the oxygen atom and the carbonyl group are in the red region, so they are interested in electrophilic attack. Also, the placement of nitrogen in blue areas indicates a more positive charge on nitrogen, which makes it susceptible to nucleophilic attack.

The chemical reactivity indices for **8a** and **8c** are shown in Table 2. According to this Table, the calculated values of total energy (E), enthalpy (H), and Gibbs free energy (G) showed that **8a** was more thermodynamically stable than **8c**. Hardness and softness parameters are obtained from HOMO and LUMO energies, as a result, compounds with a higher energy gap between HOMO and LUMO have high hardness and kinetic stability. The results shown in Table 2 stated that compound **8a** had a greater hardness value compared to compounds **8c** and Erlotinib, therefore it is more stable and less reactive.

**Table 2:** The chemical reactivity indices of **8a** and **8c** at B3LYP/6–31 + G (d, p) level of theory

Entry	E_tot_^a^	H^a^	G^a^	S^b^	ɳ^c^	σ^d^	A^c^
8a	-3946.24	-3946.24	-3946.32	162.181	2.35	0.21	1.41
8c	-3833.11	-3833.11	-3833.16	122.36	2.28	0.22	1.57
Erlotinib	-1309.54	-1309.54	-1309.63	184.46’	2.092	0.24	1.48

^a^in Hartree/particle. ^b^in cal/mol.K. ^c^in ev. ^d^in ev^− 1^

**Fig. 19:**
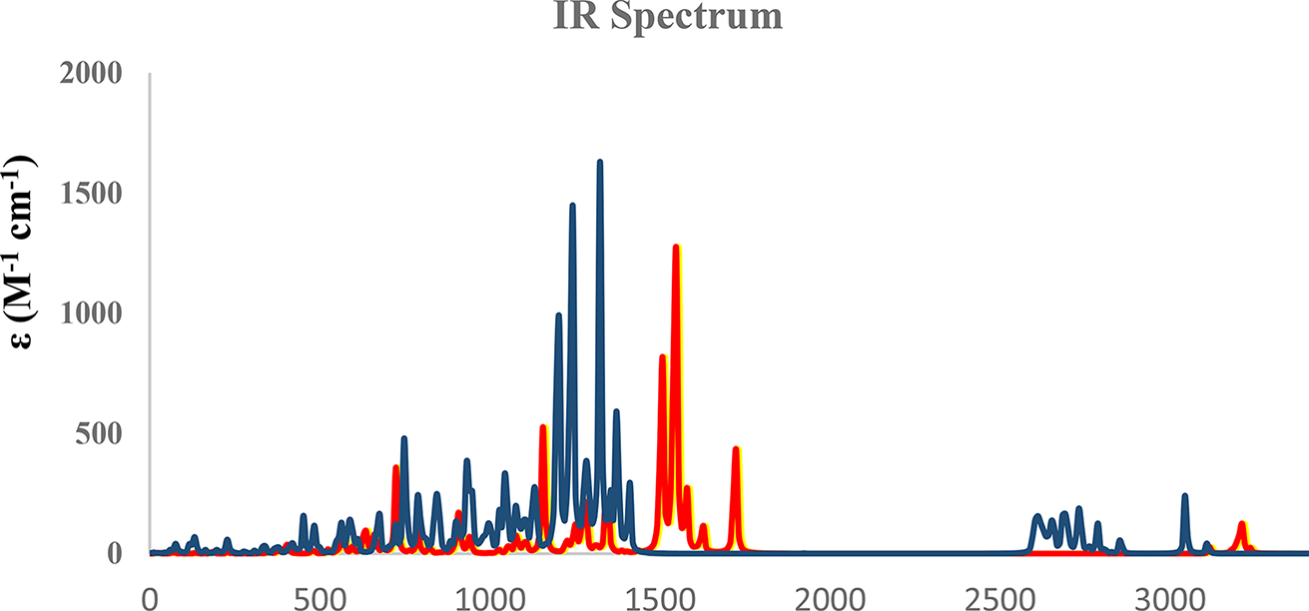
Calculated IR spectra for **8a** (red), **8c (**yellow**)** and Erlotinib (blue), at B3LYP/6–31 + G(d, p) level of theory

The theoretical analysis of the IR spectrum for **8a**, **8c**, and Erlotinib was computed by B3LYP/6–31 + G (d, p) level of theory. is indicated in Fig. 19. The IR peaks of C = C, C = N, C = O, C-N, and C-H aliphatic and aromatic excellently agree with the experimental results.

## Material and method

### Chemistry

All commercially available materials and solvents from Merck and Sigma-Aldrich Chemical Co. were used without further purification. Thin-layer chromatography (TLC) was performed on Merck silica gel (60 F_254_, Merck) plates to monitor the progress of the reactions. Melting points were determined on an electrothermal 9200 instrument and were uncorrected. The ^1^H-NMR and ^13^C-NMR spectra were recorded in CDCl_3_ on a Bruker 500 and 125 MHz spectrometer, respectively. Chemical shifts (δ) are reported in ppm scale toward tetramethylsilane (TMS) as an internal standard. Mass analyses were carried out with an Agilent Technologies (HP) mass spectrometer operating at an ionization potential of 70 eV.

### Synthesis of 5-bromoanthranilic acid (3)

Anthranilic acid **(1)** (2.74 g, 20 mmol) was dissolved in 20 mL acetonitrile. Then, a solution of N-bromosuccinimide **(2)** (3.73 g, 21 mmol) in the acetonitrile (30 mL) was added dropwise to the reaction mixture. The resulting mixture was stirred at room temperature for 2 h. Finally, the obtained precipitate was filtered, washed with acetonitrile, and dried at room temperature to afford 5-bromoanthranilic acid **(3)** with a 61.85% yield.

### Synthesis of 6-bromo-2-mercapto-3-phenylquinazolin-4(3*H*)-one (5)

A mixture of 5-bromoanthranilic acid **(3)** (2.16 g, 10 mmol), phenyl isothiocyanate **(4)** (1.8 mL, 15 mmol), and triethylamine (2 mL) in absolute ethanol (30 mL) was refluxed at 65 ˚C for 20 h. After completion of the reaction (checked by TLC), the reaction mixture was filtered and the obtained residue was recrystallized with ethanol to give the key intermediate 6-bromo-2-mercapto-3-phenylquinazolin-4(3*H*)-one **(5)** in 83.2% yield.

### Synthesis of compounds 8a-8 h

For the preparation of compounds **8a-8 h**, intermediate **5** (1 mmol) was dissolved in 15 mL DMF, and then, K_2_CO_3_ (1.2 mmol) was added over 5 min to it. In the following, various alkyl halides (1.5 mmol) or substituted benzyl bromides (1.5 mmol) were added to the reaction mixture. The resulting mixture was refluxed for 24 h. After completion of the reaction (checked by TLC), the reaction mixture was poured onto crushed ice and the obtained precipitate was filtered, dried, and recrystallized with ethanol to obtain the quinazoline-4-one derivatives **8a-8 h** in 68.5–92.3% yields [21, 22].

**1) 6-bromo-2-(butylthio)-3-phenylquinazolin-4(3** ***H*** **)-one (8a)**

White solid; yield: 74.6%, mp: 128–130 ˚C, ^1^H-NMR (500 MHz, CDCl_3_) δ (ppm): 8.37 (d, *J* = 2.0 Hz, 1H, quinazoline), 7.82 (dd, *J* = 8.5 Hz, *J* = 2.5 Hz, 1H, quinazoline), 7.58–7.56 (m, 3 H, phenyl), 7.51 (d, *J* = 8.5 Hz, 1H, quinazoline), 7.33–7.31 (m, 2 H, phenyl), 3.17 (t, *J* = 7.5 Hz, 2 H, CH_2_), 1.71–1.65 (m, 2 H, CH_2_), 1.48–1.41 (m, 2 H, CH_2_), 0.96 (t, *J* = 7.5 Hz, 3 H, CH_3_).^13^C-NMR (125 MHz, CDCl_3_) δ (ppm): 160.71, 158.48, 146.72, 137.66, 135.79, 130.01, 129.72, 129.65, 129.04, 128.07, 121.25, 118.78, 32.37, 30.64, 22.04, 13.65. MS: m/z (%): 390.1 [M^+^], 333.0 (100), 301.0 (21.95), 252.1 (22.99), 224.0 (16.23), 192.1 (26.88), 170.0 (33.48), 133.1 (21.13), 91.1 (30.40), 77.1 (59.09), 51.1 (15.61).

2) **6-bromo-2-(pentylthio)-3-phenylquinazolin-4(3** ***H*** *)-one (8b)*

White solid; yield: 68.5%, mp: 99–101 ˚C, ^1^H-NMR (500 MHz, CDCl_3_) δ (ppm): 8.37 (d, *J* = 2.5 Hz, 1H, quinazoline), 7.82 (dd, *J* = 8.5 Hz, *J* = 2.0 Hz, 1H, quinazoline), 7.58–7.57 (m, 3 H, phenyl), 7.51 (d, *J* = 8.5 Hz, 1H, quinazoline), 7.33–7.31 (m, 2 H, phenyl), 3.16 (t, *J* = 7.5 Hz, 2 H, CH_2_), 1.73–1.68 (m, 2 H, CH_2_), 1.43–1.33 (m, 4 H, CH_2_-CH_2_), 0.93 (t, *J* = 7.0 Hz, 3 H, CH_3_). ^13^C-NMR (125 MHz, CDCl_3_) δ (ppm): 160.70, 158.49, 146.74, 137.65, 135.80, 130.01, 129.72, 129.65, 129.05, 128.08, 121.26, 118.77, 32.64, 31.07, 28.26, 22.18, 13.93. MS: m/z (%): 404.2 [M^+^] (13.40), 357.2 (19.25), 333.1(100), 301.1 (23.97), 272.1 (3.30), 252.2 (18.46), 224.2 (11.04), 192.2 (16.29), 170.1 (17.95), 133.1 (7.94), 104.2 (3.28), 91.2 (10.66), 77.2 (17.35), 51.2 (2.34).

3) **2-(benzylthio)-6-bromo-3-phenylquinazolin-4(3** ***H*** **)-one (8c)**

White solid; yield: 81.9%, mp: 140–142 ˚C, ^1^H-NMR (500 MHz, CDCl_3_) δ (ppm): 8.39 (d, *J* = 2.0 Hz, 1H, quinazoline), 7.85 (dd, *J* = 9.0 Hz, *J* = 2.0 Hz, 1H, quinazoline), 7.59–7.54 (m, 5 H, phenyl), 7.40 (d, *J* = 7.0 Hz, 2 H, benzyl), 7.33–7.32 (m, 3 H, benzyl), 7.28 (d, *J* = 7.0 Hz, 1H, quinazoline), 4.43 (s, 2 H, CH_2_).^13^C-NMR (125 MHz, CDCl_3_) δ (ppm): 160.63, 157.92, 146.63, 137.78, 136.12, 135.51, 130.14, 129.79, 129.74, 129.38, 129.05, 128.59, 128.07, 127.59, 121.36, 119.03, 37.23. MS: m/z (%): 424.1 [M^+^] (14.18), 391.1 (31.87), 333.1 (14.17), 313.1 (26.29), 274.1 (3.25), 252.1 (26.32), 224.1 (12.10), 197.1 (12.48), 167.2 (100), 133.1 (10.38), 91.2 (72.98), 65.2 (14.09).

4) ** 6-bromo-2-((3-methylbenzyl)thio)-3-phenylquinazolin-4(3** ***H*** *)-one (8d)*

White solid; yield: 78.5%, mp: 101–103 ˚C, ^1^H-NMR (500 MHz, CDCl_3_) δ (ppm): 8.36 (s, 1H, quinazoline), 7.83 (d, *J* = 9.0 Hz, 1H, quinazoline), 7.56 (d, *J* = 8.5 Hz, 1H, quinazoline), 7.53–7.52 (m, 3 H, phenyl), 7.31–7.29 (m, 2 H, phenyl), 7.17 (br, 3 H, benzyl), 7.06 (d, *J* = 5.0 Hz, 1H, benzyl), 4.38 (s, 2 H, CH_2_), 2.31 (s, 3 H, CH_3_).^13^C-NMR (125 MHz, CDCl_3_) δ (ppm): 160.60, 158.04, 146.60, 138.26, 137.75, 135.77, 135.49, 130.13, 130.09, 129.75, 129.71, 129.02, 128.48, 128.37, 128.01, 126.42, 121.32, 118.99, 37.28, 21.34. MS: m/z (%): 438.1 [M^+^] (13.09), 403.1 (37.90), 346.9 (5.56), 329.0 (24.16), 313.0 (8.22), 272.0 (3.78), 252.1 (28.42), 224.1 (11.63), 197.0 (11.46), 181.1 (96.56), 165.1 (14.63), 133.0 (15.89), 105.1 (100), 77.1 (47.74), 51.1 (11.00).

5) ** 6-bromo-2-((4-methylbenzyl)thio)-3-phenylquinazolin-4(3** ***H*** *)-one (8e)*

White solid; yield: 79.4%, mp: 164–166 ˚C, ^1^H-NMR (500 MHz, CDCl_3_) δ (ppm): 8.39 (d, *J* = 2.0 Hz, 1H, quinazoline), 7.85 (dd, *J* = 8.5 Hz, *J* = 2.0 Hz, 1H, quinazoline), 7.58 (d, *J* = 8.5 Hz, 1H, quinazoline), 7.55–7.54 (m, 3 H, phenyl), 7.32–7.31 (m, 2 H, phenyl), 7.29 (d, *J* = 8.0 Hz, 2 H, benzyl), 7.12 (d, *J* = 8.0 Hz, 2 H, benzyl), 4.40 (s, 2 H, CH_2_), 2.34 (s, 3 H, CH_3_).^13^C-NMR (125 MHz, CDCl_3_) δ (ppm): 160.64, 158.07, 146.66, 137.76, 137.38, 135.53, 132.93, 130.10, 129.76, 129.72, 129.29, 129.04, 128.07, 121.35, 118.98, 37.04, 21.15. MS: m/z (%): 438.1 [M^+^] (22.25), 403.2 (40.20), 327.1 (29.40), 301.1 (6.68), 274.1 (2.65), 252.1 (27.11), 224.1 (12.05), 181.2 (100), 133.1 (7.89), 105.2 (73.17), 77.1 (18.72), 51.1 (3.13).

6) **6-bromo-2-((4-chlorobenzyl) thio)-3-phenylquinazolin-4(3** ***H*** **)-one (8f)**

White solid; yield: 83.1%, mp: 145–147 ˚C, ^1^H-NMR (500 MHz, CDCl_3_) δ (ppm): 8.38 (d, *J* = 2.0 Hz, 1H, quinazoline), 7.85 (dd, *J* = 8.5 Hz, *J* = 2.0 Hz, 1H, quinazoline), 7.56–7.55 (m, 4 H, quinazoline (1H) + phenyl (3 H)), 7.34–7.30 (m, 4 H, benzyl), 7.27 (d, *J* = 8.5 Hz, 2 H, phenyl), 4.37 (s, 2 H, CH_2_).^13^C-NMR (125 MHz, CDCl_3_) δ (ppm): 160.55, 157.51, 146.51, 137.83, 135.41, 134.93, 133.42, 130.68, 130.21, 129.81, 129.77, 129.03, 128.71, 127.99, 121.35, 119.17, 36.34. MS: m/z (%): 458.2 [M^+^] (33.12), 425.2 (50.05), 388.2 (2.50), 367.1 (4.35), 333.1 (21.56), 313.2 (14.87), 272.1 (5.20), 252.2 (38.18), 224.2 (17.28), 201.2 (100), 165.2 (15.82), 125.1 (92.49), 89.1 (24.52), 63.1 (10.47).

7) **6-bromo-2-((4-bromobenzyl) thio)-3-phenylquinazolin-4(3** ***H*** **)-one (8 g)**

White solid; yield: 92.3%, mp: 175–177 ˚C, ^1^H-NMR (500 MHz, CDCl_3_) δ (ppm): 8.35 (s, 1H, quinazoline), 7.82 (d, *J* = 8.5 Hz, 1H, quinazoline), 7.53 (br, 4 H, quinazoline (1H) + phenyl (3 H)), 7.40 (d, *J* = 8.0 Hz, 2 H, phenyl), 7.28–7.27 (m, 2 H, benzyl), 7.25 (d, *J* = 8.5 Hz, 2 H, benzyl), 4.32 (s, 2 H, CH_2_).^13^C-NMR (125 MHz, CDCl_3_) δ (ppm): 160.51, 157.44, 146.47, 137.80, 135.45, 135.37, 131.63, 130.99, 130.19, 129.78, 129.74, 129.00, 127.95, 121.49, 121.32, 119.14, 36.34. MS: m/z (%): 502.1 [M^+^] (58.74), 469.1 (87.76), 411.0 (9.19), 388.2 (6.36), 314.2 (44.69), 272.1 (8.21), 245.2 (100), 223.2 (11.03), 199.1 (25.71), 197.1 (25.26), 169.1 (71.72), 133.1 (18.06), 90.2 (37.94), 63.2 (9.51).

8) **6-bromo-2-((4-nitrobenzyl) thio)-3-phenylquinazolin-4(3** ***H*** **)-one (8 h)**

Yellow solid; yield: 87.9%, mp: 231–233 ˚C, ^1^H-NMR (500 MHz, CDCl_3_) δ (ppm): 8.37 (d, *J* = 2.0 Hz, 1H, quinazoline), 8.17 (d, *J* = 9.0 Hz, 2 H, benzyl), 7.86 (dd, *J* = 8.5 Hz, *J* = 2.5 Hz, 1H, quinazoline), 7.60–7.56 (m, 5 H, phenyl), 7.54 (d, *J* = 9.0 Hz, 1H, quinazoline), 7.33–7.30 (m, 2 H, benzyl), 4.46 (s, 2 H, CH_2_).^13^C-NMR (125 MHz, CDCl_3_) δ (ppm): 160.45, 156.76, 146.33, 144.56, 137.94, 135.25, 130.36, 130.11, 129.88, 129.84, 129.01, 127.90, 127.49, 124.27, 123.72, 121.36, 119.42, 35.97. MS: m/z (%): 469.1 [M^+^] (19.56), 436.1 (17.33), 378.0 (3.34), 333.0 (30.15), 301.1 (19.81), 274.1 (8.20), 252.1 (39.01), 212.1 (100), 199.0 (32.00), 197.0 (34.45), 170.0 (25.17), 133.1 (19.18), 106.1 (9.06), 77.1 (44.57), 51.2 (11.70).

### Cytotoxic assay

The antiproliferative activity of all the synthesized compounds **(8a-8 h)** was done by MTT (standard 3-(4,5-dimethylthiazol-yl)-2,5-diphenyl-tetrazolium bromide) assay according to our previous protocols [31, 42]. MCF-7 (breast carcinoma) and SW480 (Colorectal carcinoma) cell lines were choose [29, 43–45] and purchased from the National Cell Bank of Iran (NCBI, Pasteur Institute, Tehran, Iran). RPMI 1640 culture media with 10% fetal bovine serum (FBS) and 1% penicillin-streptomycin (Gibco, USA) were applied as culture. Trypsin/EDTA 0.5% solution (Gibco/USA) was used to harvest cells and then, the cells were seeded at a density of 1 × 10^4^ cells per well in 96-well microplates [46]. Five different concentrations of the derivatives and Cisplatin as the positive control (1 to 200 µM) were used for treatment in triplicate times. Three untreated wells were applied as the negative control. After 72 h, the media was changed by 100 µL fresh MTT solution and incubated for 4 h at 37 °C in the incubator to achieve formazan purple crystals [47]. Finally, the media was removed and 150 µL of DMSO was added and incubated at 37 °C in the dark for 10 min to dissolve the crystals. A Microplate ELISA reader was applied to read the absorbance of individual wells at 490 nm. Analysis of the data was obtained by Excel 2016 and Curve Expert 1.4. The data was expressed as the mean ± SD for each analysis. GraphPad Prism software was used to perform one-way ANOVA statistical analyses, followed by Tukey’s multiple comparison test.

### Molecular docking study

The crystal structure of the EGFR target was downloaded from the RCSB protein data bank site (PDB ID: 1M17) [48]. AutoDock Vina was used to run the molecular docking procedure. The structure of compounds was minimized in terms of energy and converted to pdbqt format. A grid box of 70 × 70 × 70 Å and an exhaustiveness of 100 were set for docking analysis. To visualize the interaction and orientation of the compounds, the Discovery Studio 2016 client was used.

### Molecular dynamic simulation

The Gromacs molecular dynamics package on a Centos Linux server equipped with GPUs was used to perform the molecular dynamics simulation of compounds **8a**, **8c** and native ligand (Erlotinib) in complex with EGFR protein kinase (Pdb: 1M17) ), as well as the **8a** compound with EGFR-mutated protein (3W2Q). The Amber99sb force field was employed to define the atom types and simulate the dynamics of the system. ACPYPE webserver was used to generate the compounds **8a**, **8c** and native ligand topology parameters. An octahedral box was defined around the solute (compounds **8a**, **8c** and Erlotinib) in a complex with EGFR and EGFR-mutated kinase. TIP3P water molecules were added to solvate the protein complex model, ensuring it was surrounded by water. The appropriate number of Na^+^ ions replaced water molecules to ensure the system neutralization. The system was subjected to NVT heating. Restraints were applied to the solute (compounds **8a**, **8c** and Erlotinib) to prevent large conformational changes during the initial equilibration phase. The particle-mesh Ewald (PME) algorithm was used to efficiently handle long-range electrostatic interactions. The system’s pressure was stabilized at an average pressure of 1 atm. This step ensures that the density of water molecules reaches an equilibrium state during the equilibration phase. The system was equilibrated under the NPT ensemble (constant Number of particles, Pressure, and Temperature) for 500 ps. In general, molecular dynamics simulations require to be adequately long to be able to draw reliable conclusions. The production MD run was performed during 100 ns to obtain the best equilibration point of the system with favorable temperature and pressure. After the MD run was completed, the trajectory (the sequence of atomic coordinates over time) was corrected for periodic boundary conditions. To determine the equilibrium time range within the MD simulation, the root-mean-square deviation (RMSD) was considered. RMSD measures the deviation of atomic positions in each snapshot of the trajectory from a reference frame, typically the starting structure [49].

### DFT analysis

Density functional theory was used to investigate the reactivity descriptors of the compounds with the highest (**8a**) and lowest (**8c**) biological activity at the B3LYP/6–31 + G (d, p) level of theory. The molecular orbitals (HOMO and LUMO), electrostatic surface potential energy, and thermochemical parameters were also studied in detail.

## Conclusion

In this study, a series of quinazoline-4(3*H*)-one derivatives were synthesized and evaluated as cytotoxic agents. The SARs were presented by analyzing the impact of varying substitutions on SH and phenyl fragments. 6-bromo-2-(butylthio)-3-phenylquinazolin-4(3*H*)-one (**8a)** displayed the best cytotoxic activity and is about 2 times more potent than Erlotinib in MCF-7 cell line. Cytotoxic results on normal cell line showed that all of the compounds had appropriate selectivity between tumorigenic and non-tumorigenic cell lines. Molecular docking studies against EGFR and EGFR-mutated were applied to determine the binding conformation of all derivatives. Based on these values, it can be mentioned that compound **8a** was located in the binding pocket of EGFR and EGFR-mutated and bonded strongly to both receptors. The DFT analysis was performed for more active and less active compounds. The reactivity descriptors, the energy gap between HOMO and LUMO revealed that **8a** is more stable than **8c**. Also, a good agreement between theoretical and experimental IR spectra was observed. Finally, the outcomes obtained from the MD simulation, which encompassed parameters such as RMSD, RMSF, Rg, and the count of hydrogen bonds demonstrated the stability of compound **8a, 8c** and Erlotinib complexes within the active site of EGFR and EGFR-mutated enzymes throughout the entire simulation duration.

### Electronic supplementary material

Below is the link to the electronic supplementary material.

Supplementary Material 1

## Data Availability

The data sets used and analyzed during the current study are available from the corresponding author on reasonable request. We have presented all data in the form of Tables and Figure. The PDB code (1M17) was retrieved from protein data bank (www.rcsb.org). https://www.rcsb.org/structure/1m17.

## Abbreviations

EGFR: Epidermal Growth Factor Receptor
MDR: Multidrug Resistance
ADME: Adsorption, Distribution, Metabolism and Discretion
Rg: Radius of gyration
RMSD: Root Mean Square Deviation
RMSF: Root Mean Square Fluctuation
TPSA: Total polar surface area
TEA: Triethylamine
TK: Tyrosine kinase
MTT: 3-(4, 5 dimethylthiazol-yl)-2,5-diphenyl-tetrazolium bromide

## References

[1] Dhuguru J, Ghoneim OA (2022). Quinazoline based HDAC dual inhibitors as potential Anti-cancer agents. Molecules.

[2] Wang Q (2022). Novel approaches for the solid-phase synthesis of Dihydroquinazoline-2 (1H)-One derivatives and biological evaluation as potential Anticancer agents. Molecules.

[3] Bansal R, Malhotra A (2021). Therapeutic progression of quinazolines as targeted chemotherapeutic agents. Eur J Med Chem.

[4] Khalifa MM (2022). Topo II inhibition and DNA intercalation by new phthalazine-based derivatives as potent anticancer agents: design, synthesis, anti-proliferative, docking, and in vivo studies. J Enzyme Inhib Med Chem.

[5] Niu Z (2022). Discovery of Novel quinazoline derivatives as potent Antitumor agents. Molecules.

[6] Ferlay J (2021). Cancer statistics for the year 2020: an overview. Int J Cancer.

[7] Behranvand N (2022). Chemotherapy: a double-edged sword in cancer treatment. Cancer Immunol Immunother.

[8] Alanazi AM (2016). Synthesis, antitumor and antimicrobial activity of some new 6-methyl-3-phenyl-4 (3 H)-quinazolinone analogues: in silico studies. J Enzyme Inhib Med Chem.

[9] Dickens E, Ahmed S (2021). Principles of cancer treatment by chemotherapy. Surg (Oxford).

[10] Lin S (2017). Design, synthesis and biological evaluation of quinazoline–phosphoramidate mustard conjugates as anticancer drugs. Eur J Med Chem.

[11] Hawash M (2021). Synthesis, chemo-informatics, and anticancer evaluation of fluorophenyl-isoxazole derivatives. Open Chem.

[12] Hawash M (2022). Design and synthesis of novel substituted indole-acrylamide derivatives and evaluation of their anti-cancer activity as potential tubulin-targeting agents. J Mol Struct.

[13] Hawash M (2022). Recent advances of tubulin inhibitors targeting the colchicine binding site for cancer therapy. Biomolecules.

[14] Haghighijoo Z (2022). Therapeutic potential of quinazoline derivatives for Alzheimer’s disease: a comprehensive review. Eur J Med Chem.

[15] Zhang Y (2019). Enrichment of novel quinazoline derivatives with high antitumor activity in mitochondria tracked by its self-fluorescence. Eur J Med Chem.

[16] Khabnadideh S, Sadeghian S. A review on current synthetic methods of 4-aminoquinazoline derivatives. J Chem. 2022;2022.

[17] Alqahtani AS (2022). Cytotoxicity of newly synthesized quinazoline–sulfonamide derivatives in human leukemia cell lines and their effect on hematopoiesis in zebrafish embryos. Int J Mol Sci.

[18] Wang C-J (2021). Discovery of penipanoid C-inspired 2-(3, 4, 5-trimethoxybenzoyl) quinazolin-4 (3H)-one derivatives as potential anticancer agents by inhibiting cell proliferation and inducing apoptosis in hepatocellular carcinoma cells. Eur J Med Chem.

[19] Alagarsamy V (2018). An overview of quinazolines: pharmacological significance and recent developments. Eur J Med Chem.

[20] Chang J (2017). Development of a series of novel 4-anlinoquinazoline derivatives possessing quinazoline skeleton: design, synthesis, EGFR kinase inhibitory efficacy, and evaluation of anticancer activities in vitro. Eur J Med Chem.

[21] Khodair AI, Alsafi MA, Nafie MS (2019). Synthesis, molecular modeling and anti-cancer evaluation of a series of quinazoline derivatives. Carbohydr Res.

[22] Abuelizz HA (2017). Synthesis and anticancer activity of new quinazoline derivatives. Saudi Pharm J.

[23] Le Y (2020). Design, synthesis and in vitro biological evaluation of quinazolinone derivatives as EGFR inhibitors for antitumor treatment. J Enzyme Inhib Med Chem.

[24] Allam HA (2020). Design and synthesis of some new 2, 4, 6-trisubstituted quinazoline EGFR inhibitors as targeted anticancer agents. Bioorg Chem.

[25] Tu Y (2017). Design, synthesis, and docking studies of quinazoline analogues bearing aryl semicarbazone scaffolds as potent EGFR inhibitors. Bioorg Med Chem.

[26] Awad MK (2018). Design, synthesis, molecular modeling, and biological evaluation of novel α-aminophosphonates based quinazolinone moiety as potential anticancer agents: DFT, NBO and vibrational studies. J Mol Struct.

[27] Ghorab MM (2016). Design, synthesis and anticancer evaluation of novel quinazoline-sulfonamide hybrids. Molecules.

[28] Zayed MF (2018). Design, synthesis, cytotoxic evaluation and molecular docking of new fluoroquinazolinones as potent anticancer agents with dual EGFR kinase and tubulin polymerization inhibitory effects. Int J Mol Sci.

[29] Zare S (2023). Design, synthesis, computational study and cytotoxic evaluation of some new quinazoline derivatives containing pyrimidine moiety. Sci Rep.

[30] Ataollahi E et al. Novel quinazolinone derivatives as Anticancer agents: design, synthesis, Biological evaluation and computational studies. J Mol Struct. 2023:136622.

[31] Emami L (2020). Design, synthesis, molecular simulation, and biological activities of novel quinazolinone-pyrimidine hybrid derivatives as dipeptidyl peptidase-4 inhibitors and anticancer agents. New J Chem.

[32] Emami L (2022). Synthesis, biological evaluation, and computational studies of some novel quinazoline derivatives as anticancer agents. BMC Chem.

[33] Ye L (2019). Discovery of aminopyridine-containing spiro derivatives as EGFR mutations inhibitors. J Enzyme Inhib Med Chem.

[34] Arshia AH (2021). De novo design of novel protease inhibitor candidates in the treatment of SARS-CoV-2 using deep learning, docking, and molecular dynamic simulations. Comput Biol Med.

[35] Hawash M (2022). Anticancer activity of thiophene carboxamide derivatives as CA-4 biomimetics: synthesis, biological potency, 3D spheroid model, and molecular dynamics simulation. Biomimetics.

[36] Emami L (2022). Novel n-substituted isatin‐ampyrone schiff bases as a new class of antiproliferative agents: design, synthesis, molecular modeling and in vitro cytotoxic activity. J Heterocycl Chem.

[37] Knapp B, Ospina L, Deane CM (2018). Avoiding false positive conclusions in molecular simulation: the importance of replicas. J Chem Theory Comput.

[38] Hawash M (2023). New Thiazole Carboxamide derivatives as COX inhibitors: design, synthesis, Anticancer Screening, in Silico Molecular Docking, and ADME Profile studies. ACS Omega.

[39] Hirao H (2011). Which DFT functional performs well in the calculation of methylcobalamin? Comparison of the B3LYP and BP86 functionals and evaluation of the impact of empirical dispersion correction. J Phys Chem A.

[40] Kavitha T, Velraj G (2016). Structural, spectroscopic (FT-IR, FT-Raman, NMR) and computational analysis (DOS, NBO, Fukui) of 3, 5-dimethylisoxazole and 4-(chloromethyl)-3, 5-dimethylisoxazole: a DFT study. J Theoretical Comput Chem.

[41] Bruna-Larenas T, Gomez-Jeria JS. A DFT and semiempirical model-based study of opioid receptor affinity and selectivity in a group of molecules with a morphine structural core. Int J Med Chem. 2012;2012. 10.1155/2012/682495 PMC4207423 25379287

[42] Su S, et al. Novel penta-1, 4-diene-3-one derivatives containing quinazoline and oxime ether fragments: design, synthesis and bioactivity. Bioorganic Med Chem. 2021:115999. 10.1016/j.bmc.2021.115999 33444848

[43] Faraj FL et al. Synthesis, characterization, and anticancer activity of new quinazoline derivatives against MCF-7 cells. Sci World J. 2014:2014. 10.1155/2014/212096 PMC4274848 25548779

[44] Bathula R (2020). Evaluation of antitumor potential of synthesized novel 2-substituted 4-anilinoquinazolines as quinazoline-pyrrole hybrids in MCF-7 human breast cancer cell line and A-549 human lung adenocarcinoma cell lines. Future J Pharm Sci.

[45] Zahedifard M (2015). Synthesis, characterization and apoptotic activity of quinazolinone Schiff base derivatives toward MCF-7 cells via intrinsic and extrinsic apoptosis pathways. Sci Rep.

[46] Faghih Z (2019). Synthesis of some novel dibromo-2-arylquinazolinone derivatives as cytotoxic agents. Res Pharm Sci.

[47] Hashemi S (2021). Two new cytotoxic ursane triterpenoids from the aerial parts of Salvia Urmiensis Bunge. Fitoterapia.

[48] Amelia T (2022). Computational prediction of Resistance Induced Alanine-Mutation in ATP site of epidermal growth factor receptor. Int J Mol Sci.

[49] Zare F (2024). A combination of virtual screening, molecular dynamics simulation, MM/PBSA, ADMET, and DFT calculations to identify a potential DPP4 inhibitor. Sci Rep.

