# Supplementary material for: 6-Bromo quinazoline derivatives as cytotoxic agents: design, synthesis, molecular docking and MD simulation
Source: BMC Chem. 2024 Jul 4;18(1):125. doi: 10.1186/s13065-024-01230-2 (PMC11225515; doi:10.1186/s13065-024-01230-2)
Supplement: Supplementary file 1 — Supplementary Material 1 [file 13065_2024_1230_MOESM1_ESM.docx]

**6-Bromo Quinazoline Derivatives as Cytotoxic agents: Design, Synthesis, Molecular Docking and MD Simulation**

Leila Emami^1^, Maryam Hassani^2^, Pegah Mardaneh^2,3^, Fateme Zare^1^, Maryam saeedi^2^, Mina Emami^1^, Soghra Khabnadideh^1,2*^, Sara Sadeghian^2^^[[1]](#footnote-1)^*

*^1^Pharmaceutical Sciences Research Center, Shiraz University of Medical Sciences, Shiraz, Iran.*

*^2^Department of Medicinal Chemistry, Faculty of Pharmacy, Shiraz University of Medical Sciences, Shiraz, Iran.*

*^3^Medicinal and Natural Products Chemistry Research Center, Shiraz University of Medical*

*Sciences, Shiraz, Iran*


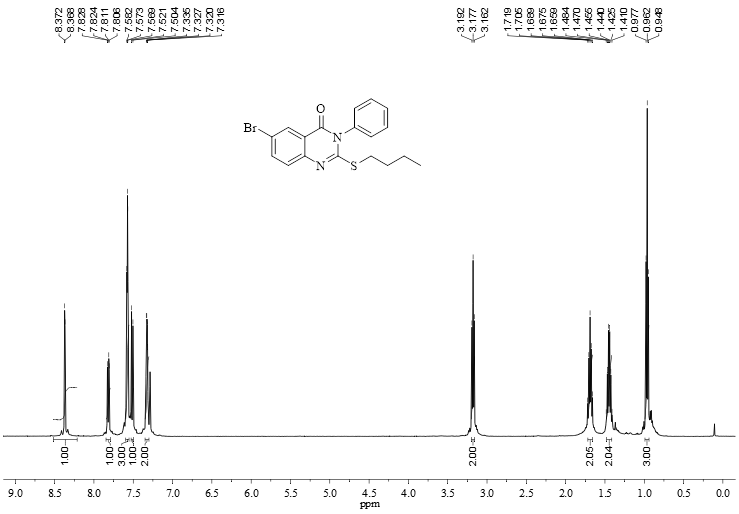


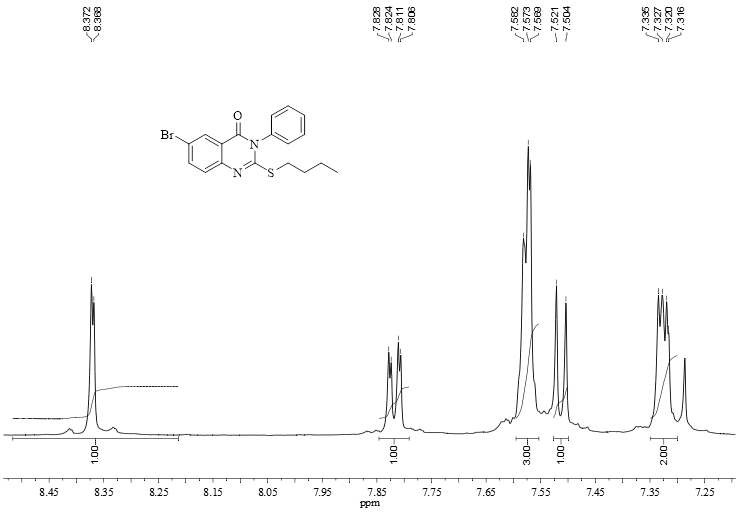


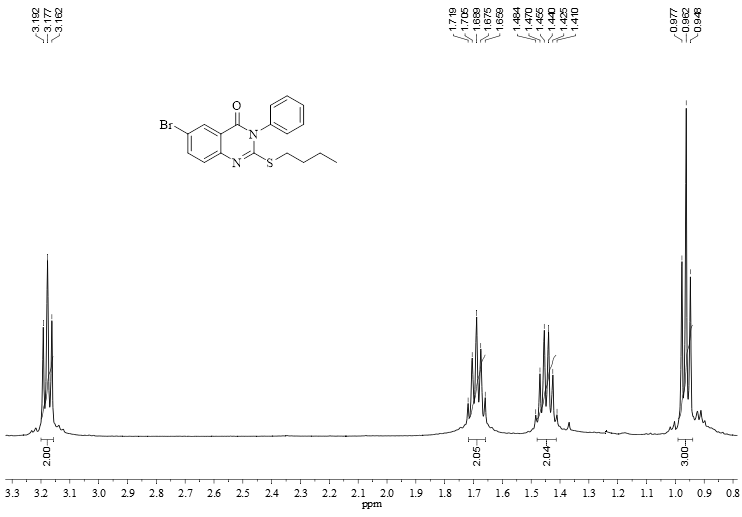


**Figure S1.** ^1^H-NMR spectrum of compound **8a.**


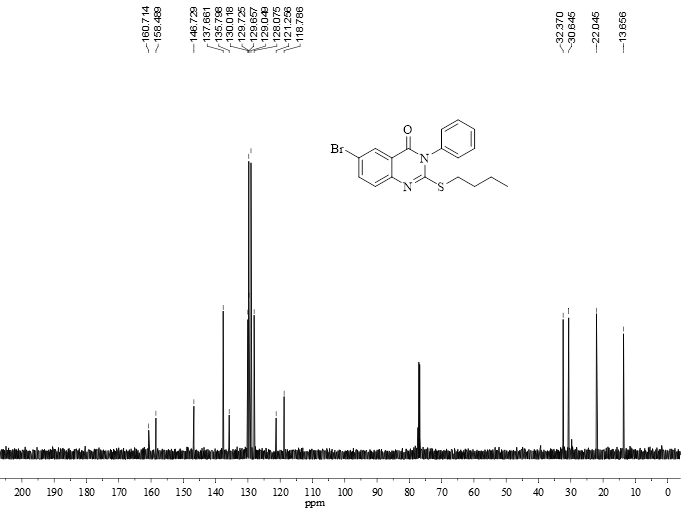


**Figure S2.** ^13^C-NMR spectrum of compound **8a.**


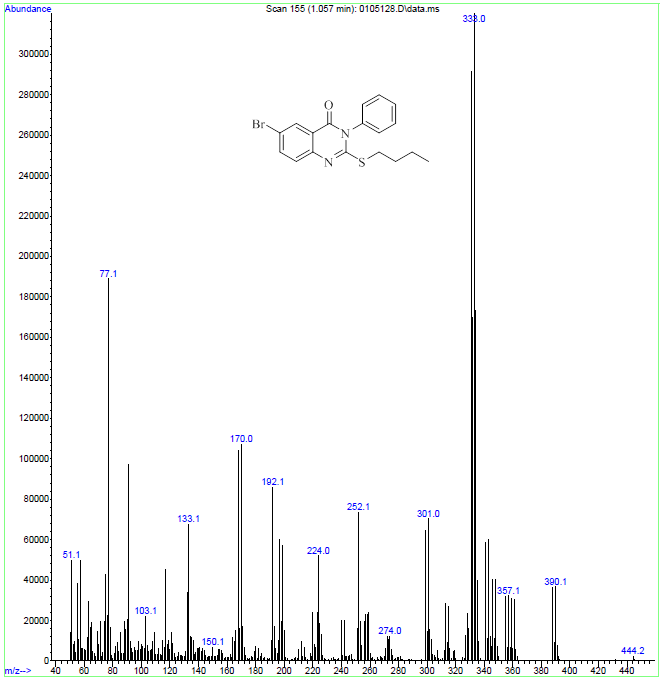


**Figure S3.** Mass spectrum of compound **8a.**


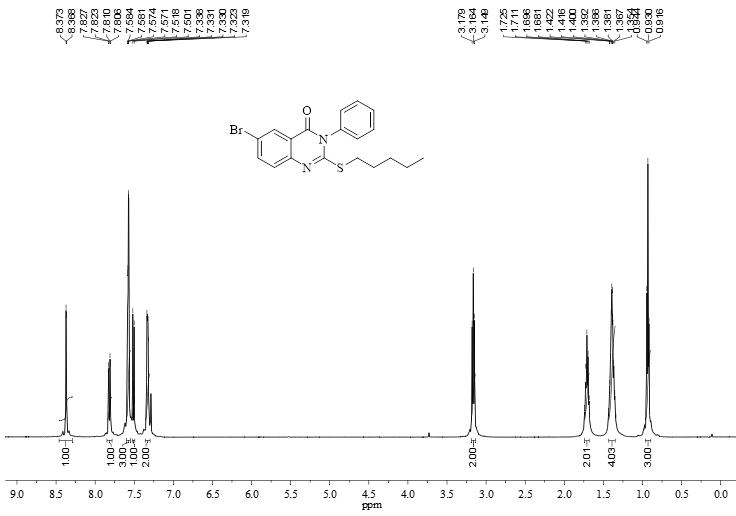


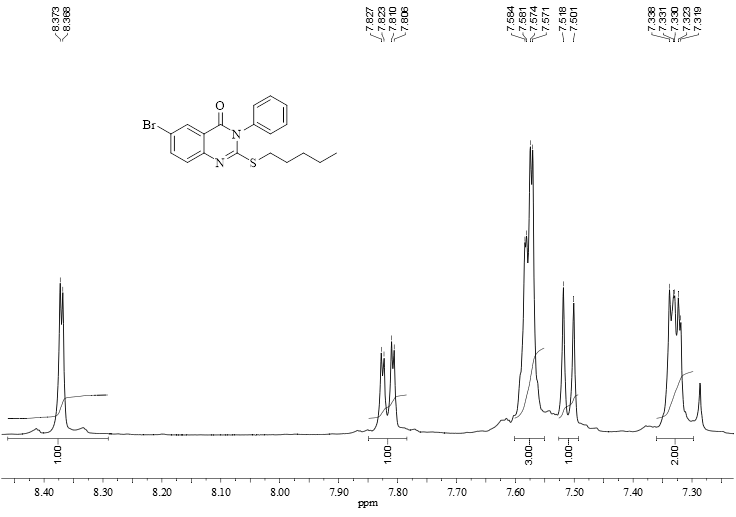


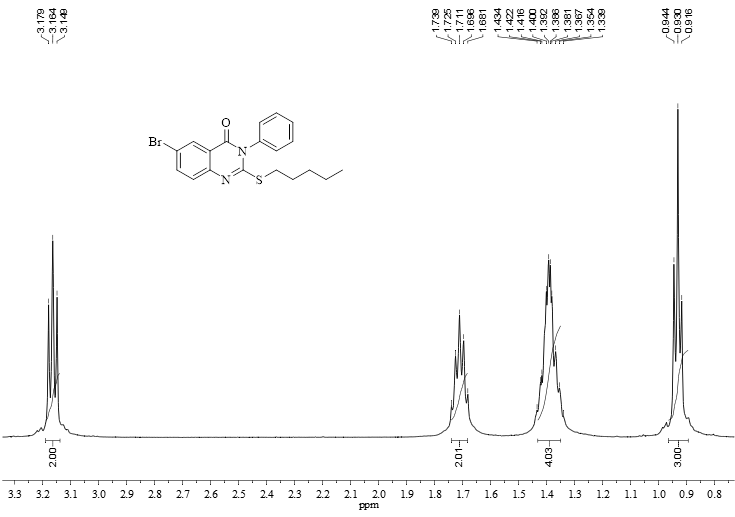


**Figure S4.** ^1^H-NMR spectrum of compound **8b.**


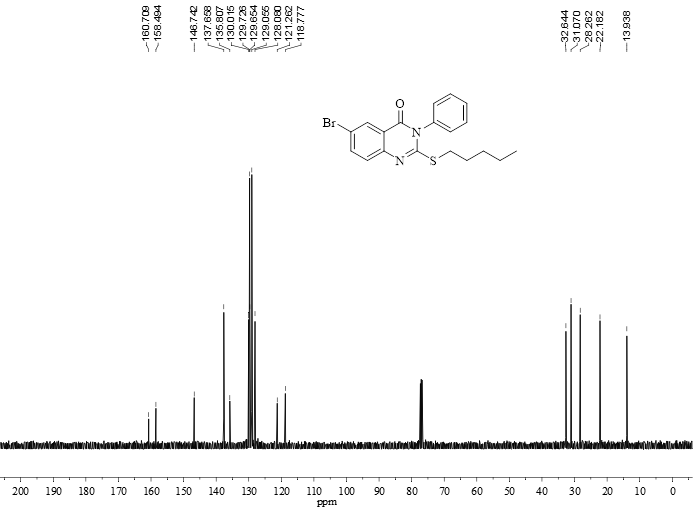


**Figure S5.** ^13^C-NMR spectrum of compound **8b.**


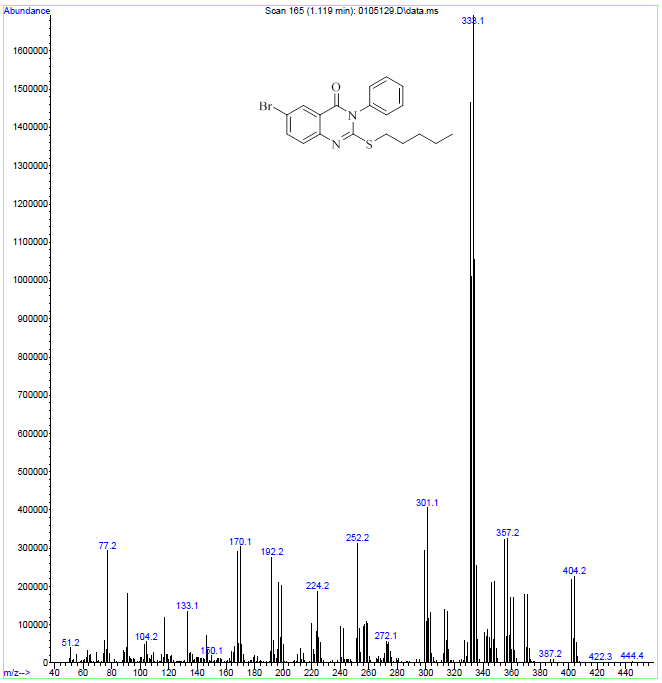


**Figure S6.** Mass spectrum of compound **8b.**


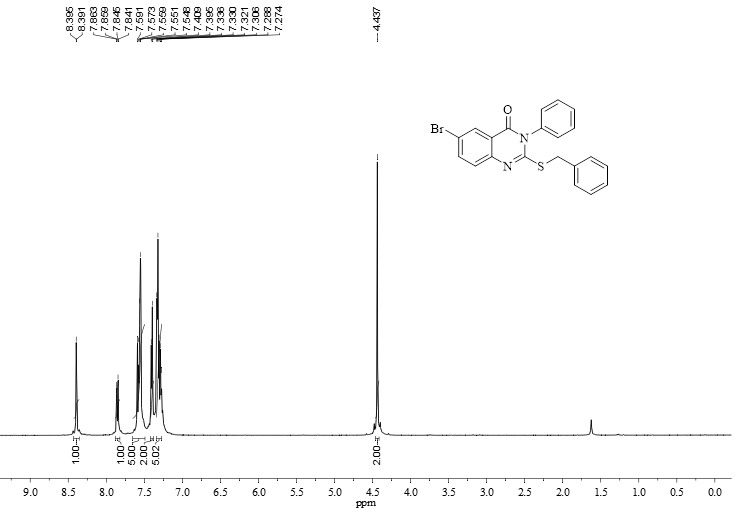


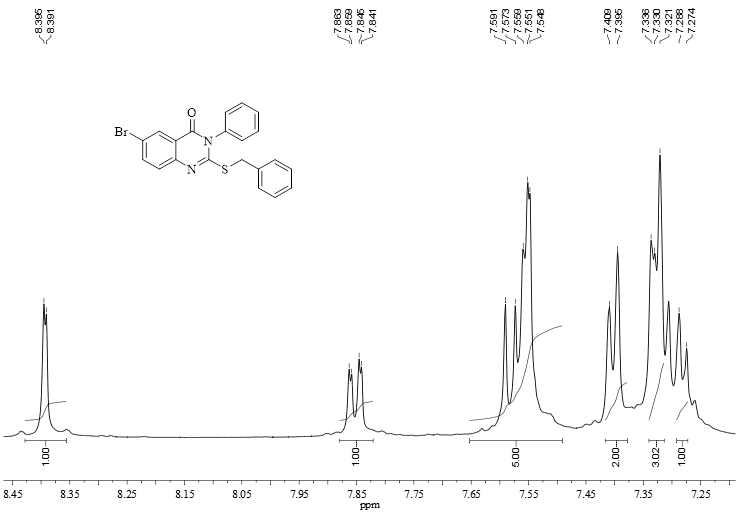


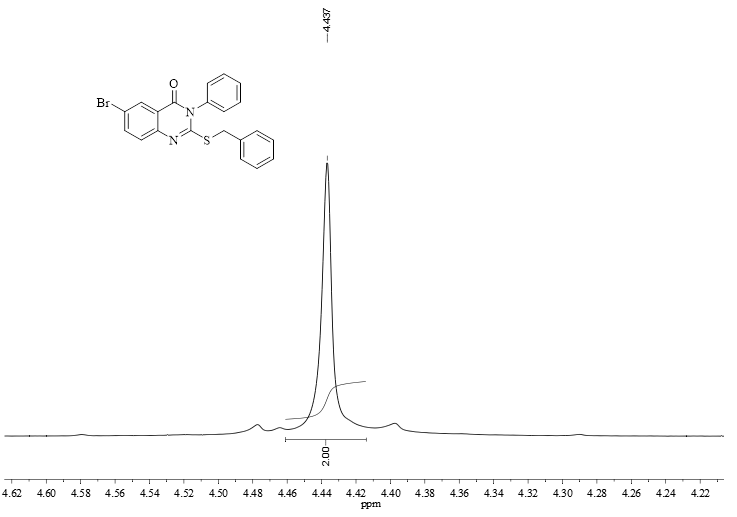


**Figure S7.** ^1^H-NMR spectrum of compound **8c.**


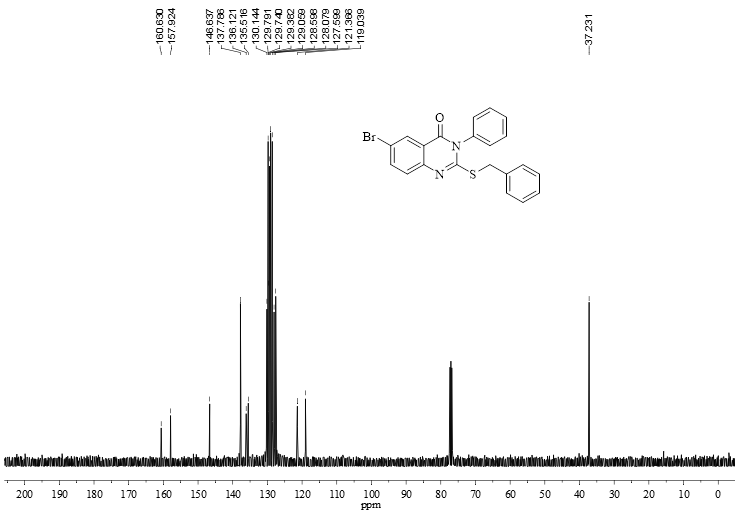


**Figure S8.** ^13^C-NMR spectrum of compound **8c.**


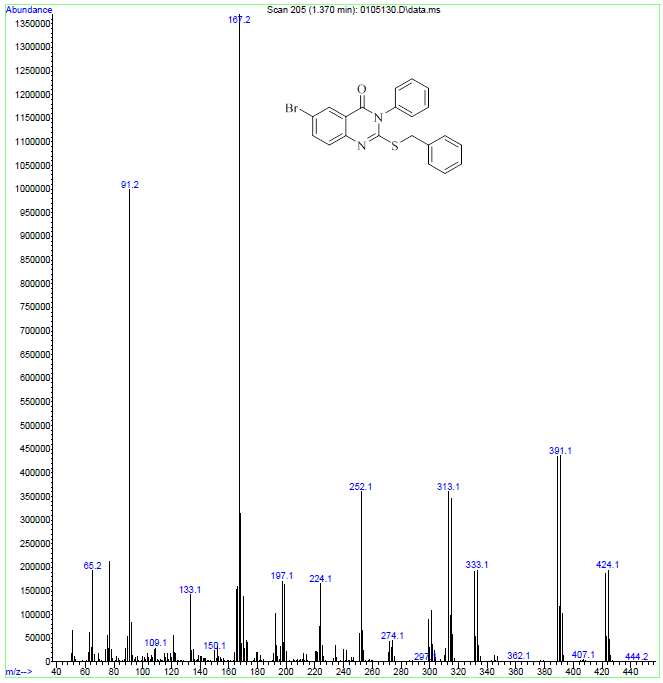


**Figure S9.** Mass spectrum of compound **8c.**


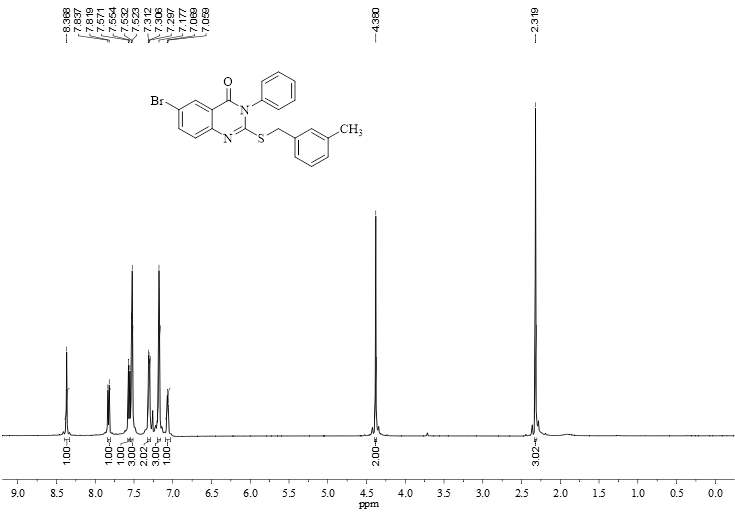


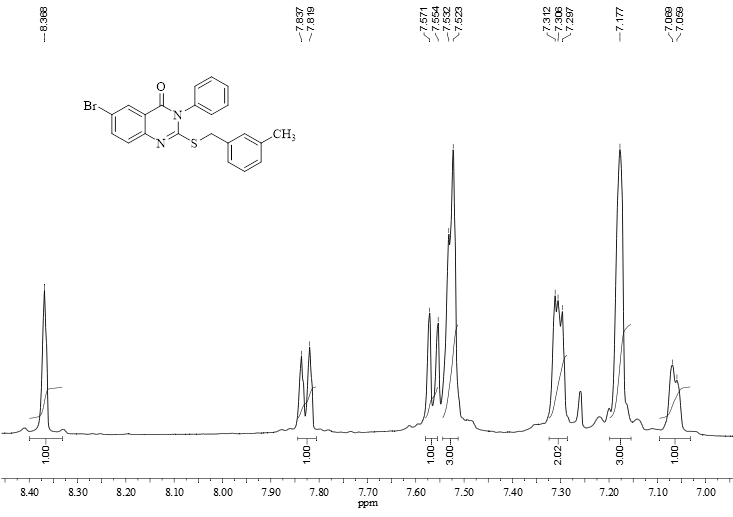


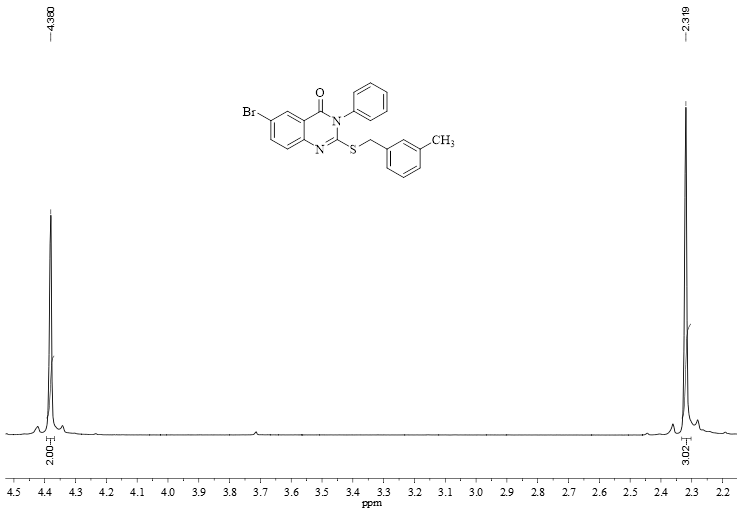


**Figure S10.** ^1^H-NMR spectrum of compound **8d.**


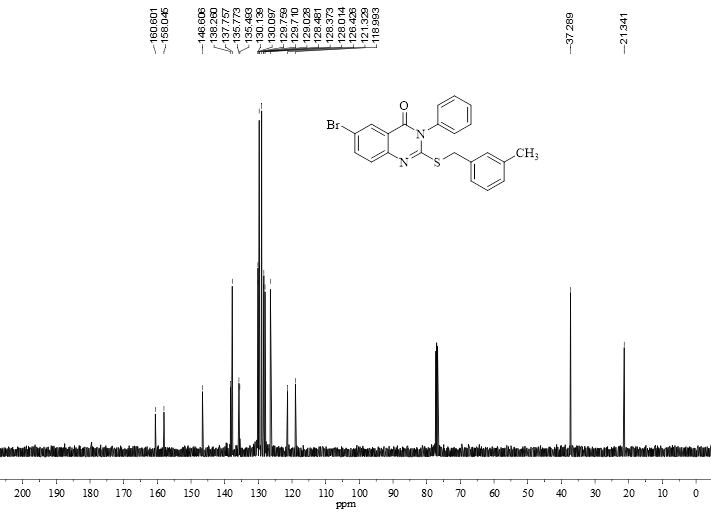


**Figure S11.** ^13^C-NMR spectrum of compound **8d.**


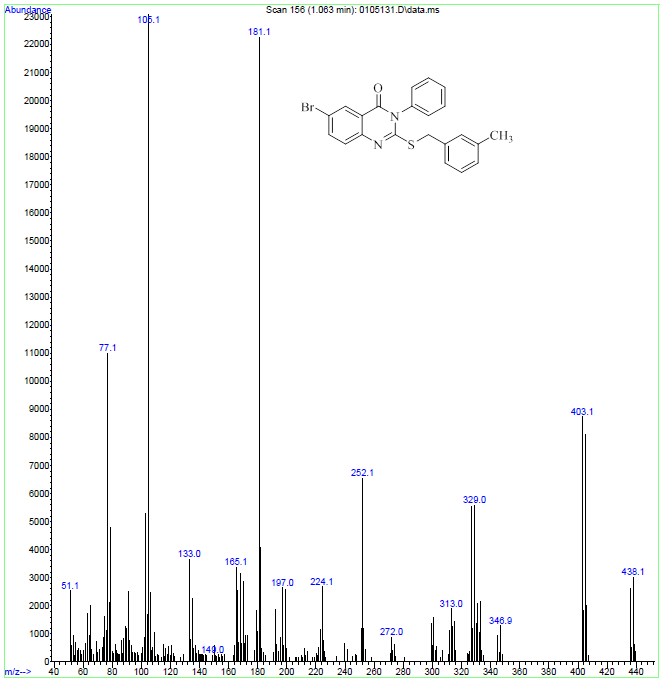


**Figure S12.** Mass spectrum of compound **8d.**


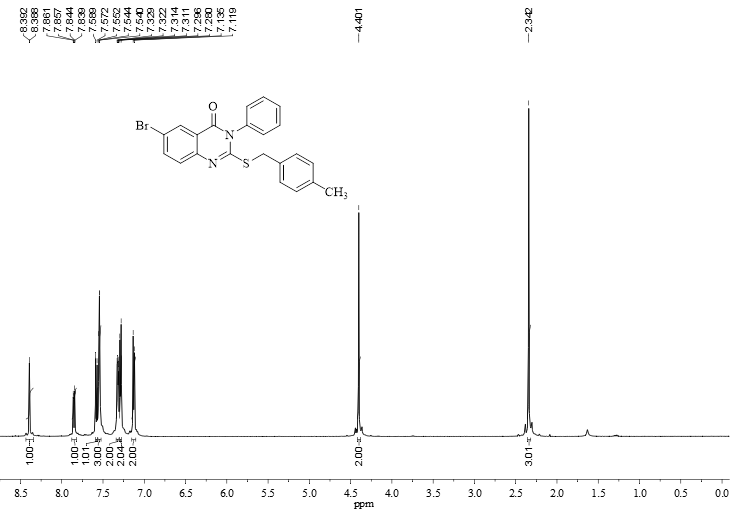


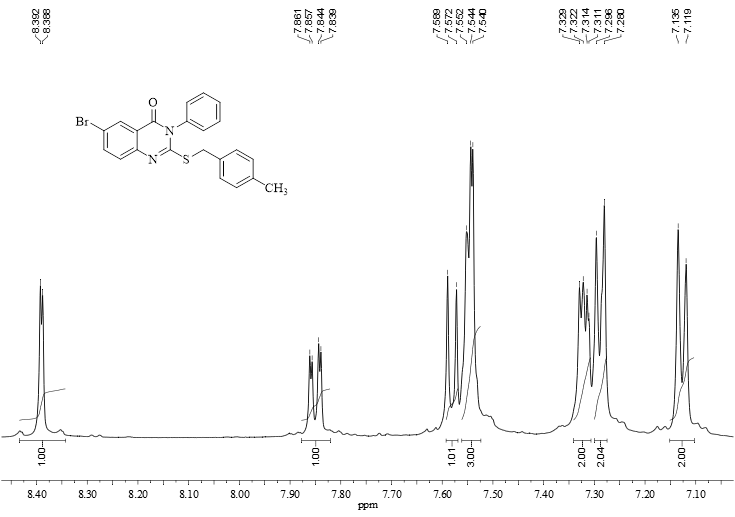


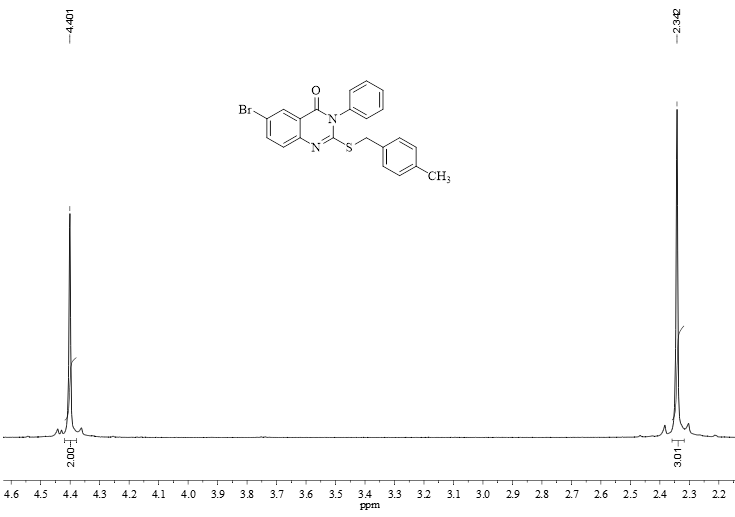


**Figure S13.** ^1^H-NMR spectrum of compound **8e.**


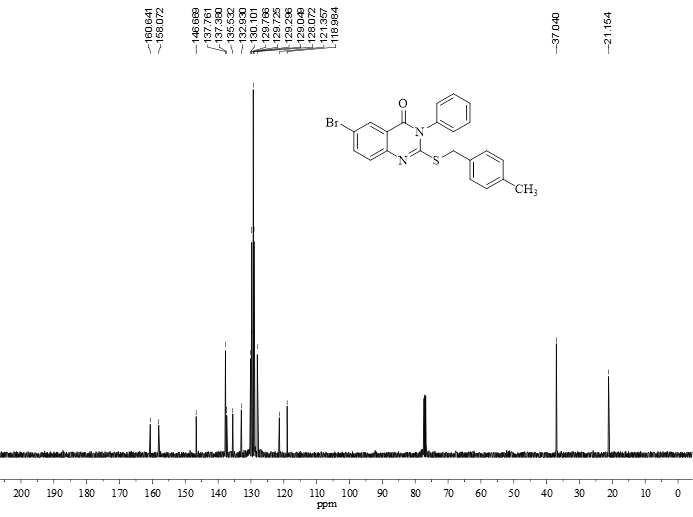


**Figure S14.** ^13^C-NMR spectrum of compound **8e.**


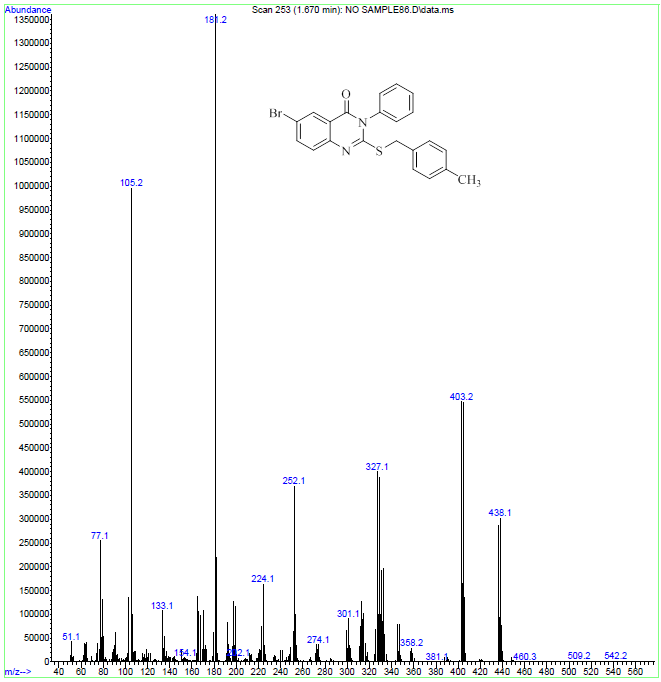


**Figure S15.** Mass spectrum of compound **8e.**


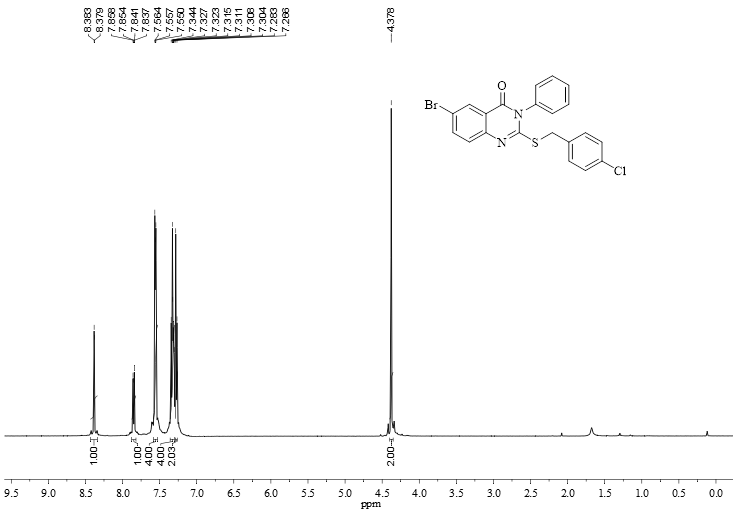


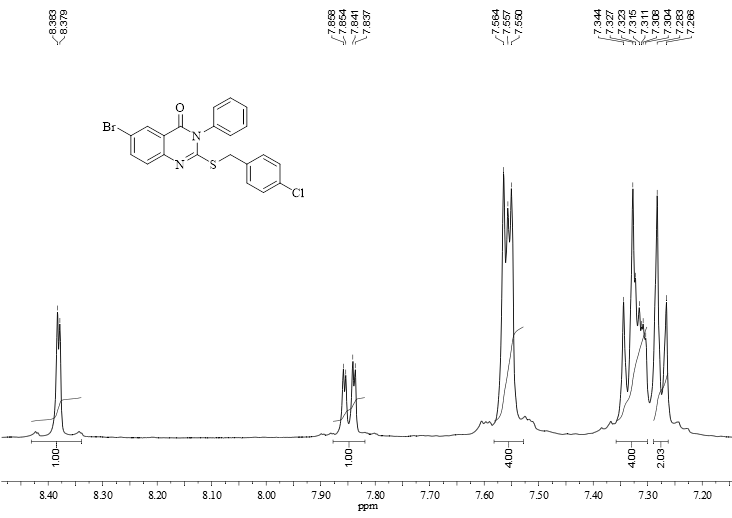


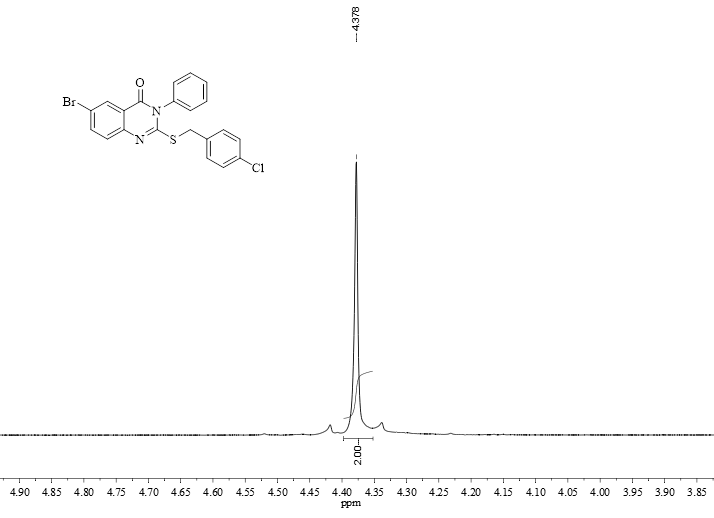


**Figure S16.** ^1^H-NMR spectrum of compound **8f.**


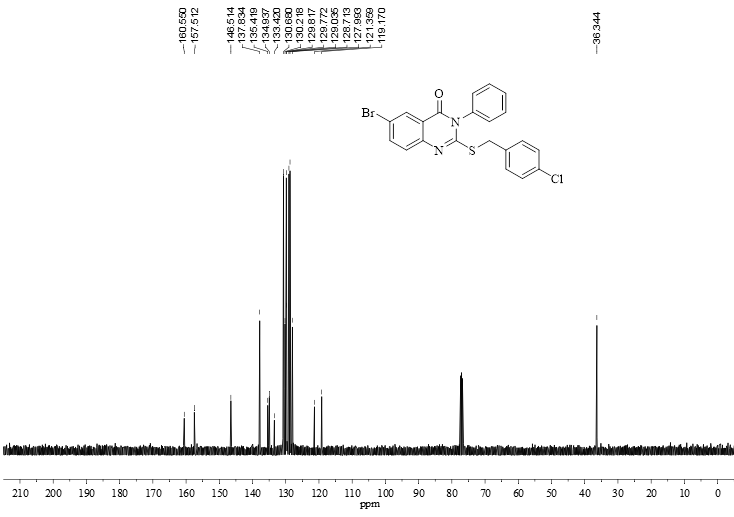


**Figure S17.** ^13^C-NMR spectrum of compound **8f.**


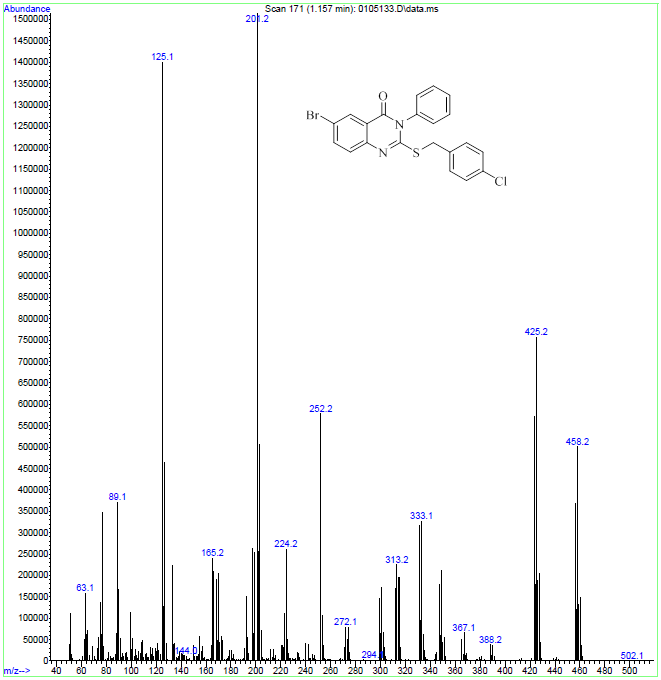


**Figure S18.** Mass spectrum of compound **8f.**


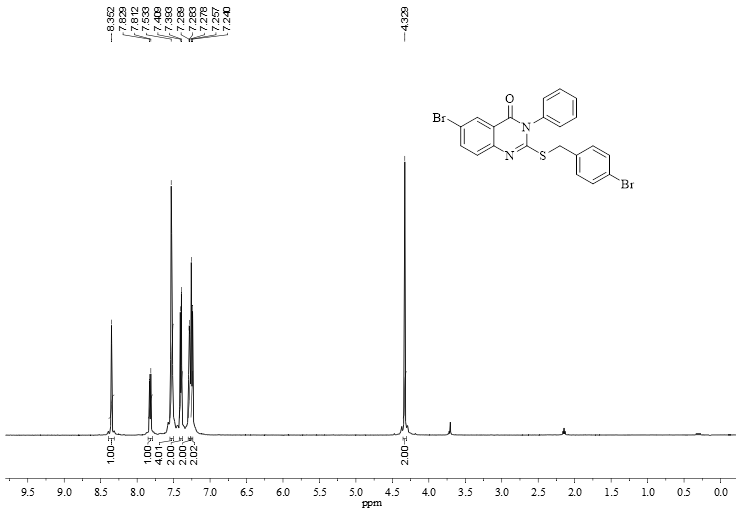


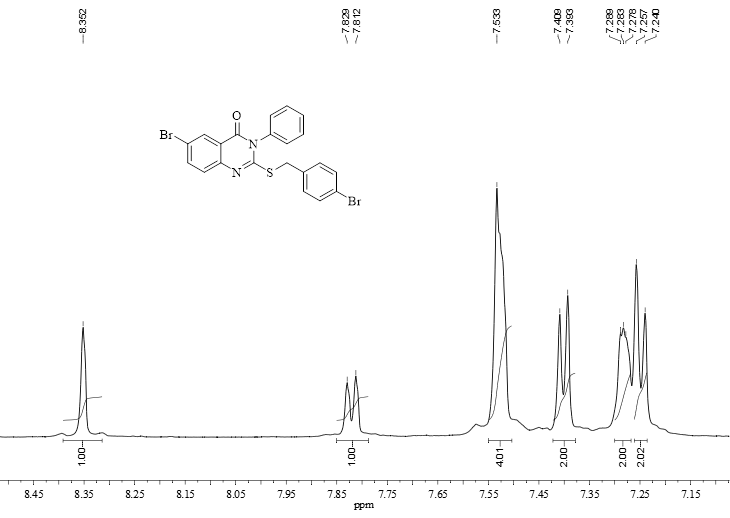


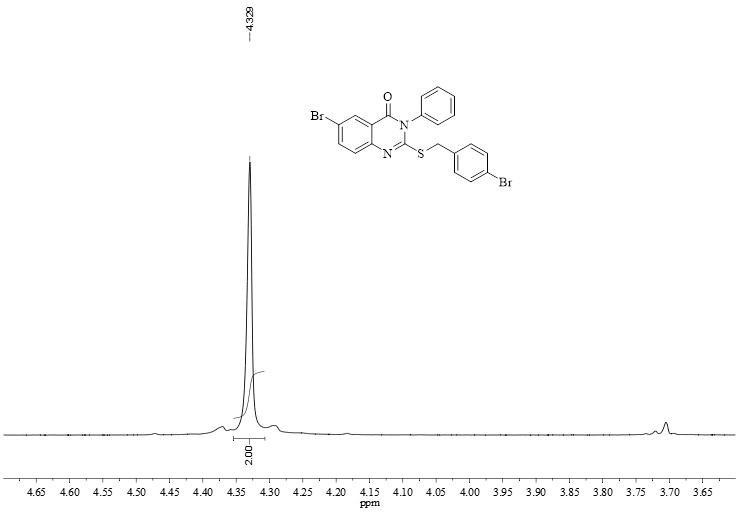


**Figure S19.** ^1^H-NMR spectrum of compound **8g.**


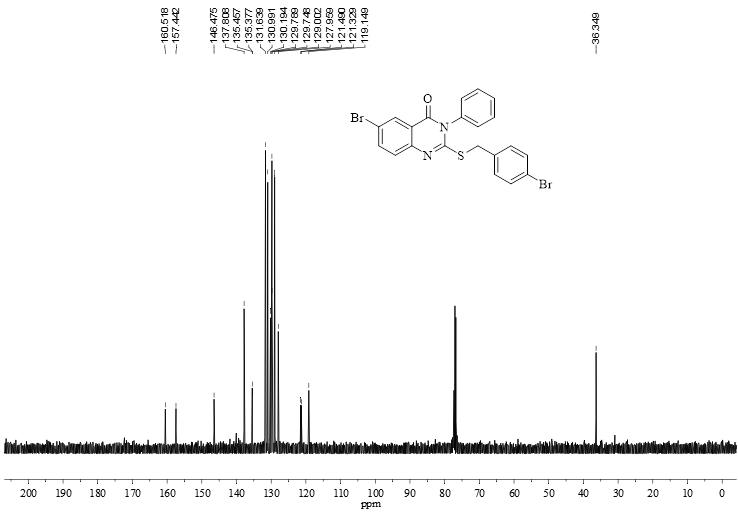


**Figure S20.** ^13^C-NMR spectrum of compound **8g.**


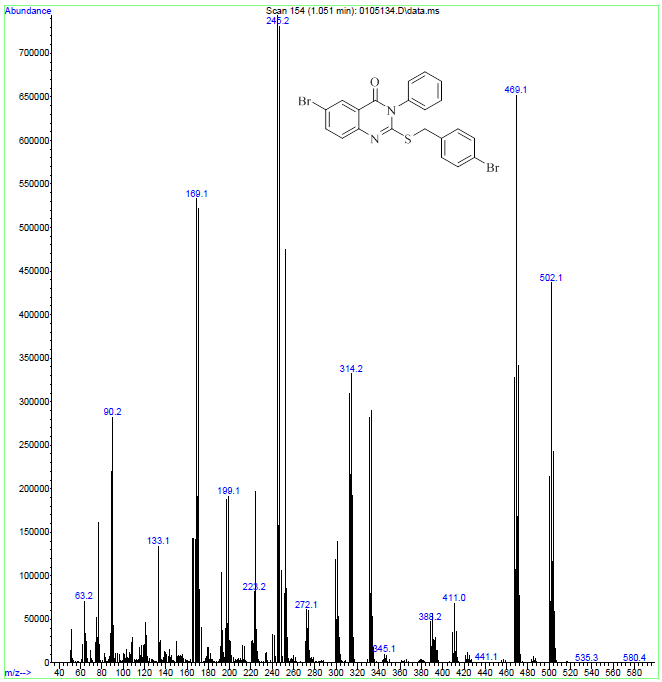


**Figure S21.** Mass spectrum of compound **8g.**


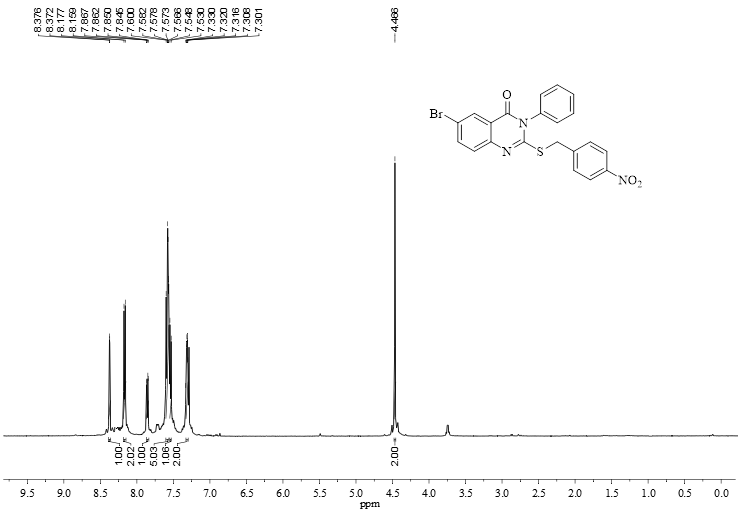


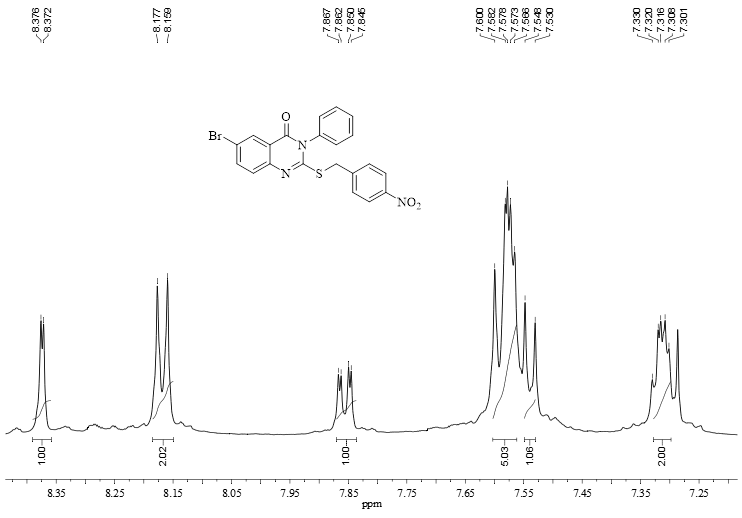


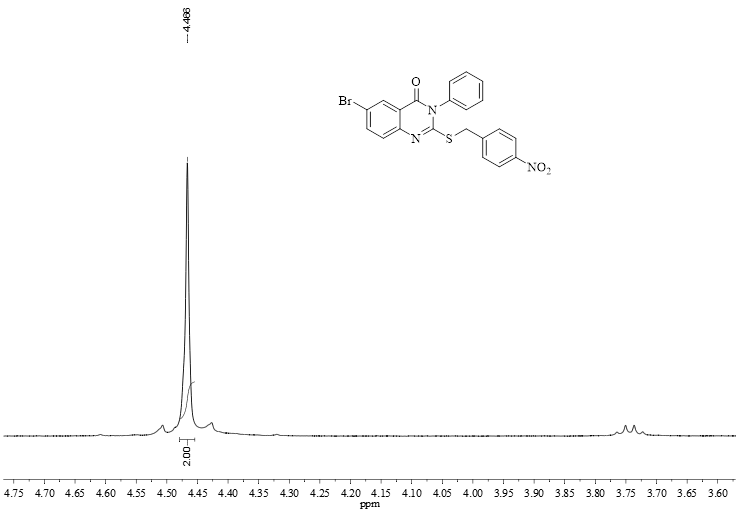


**Figure S22.** ^1^H-NMR spectrum of compound **8h.**


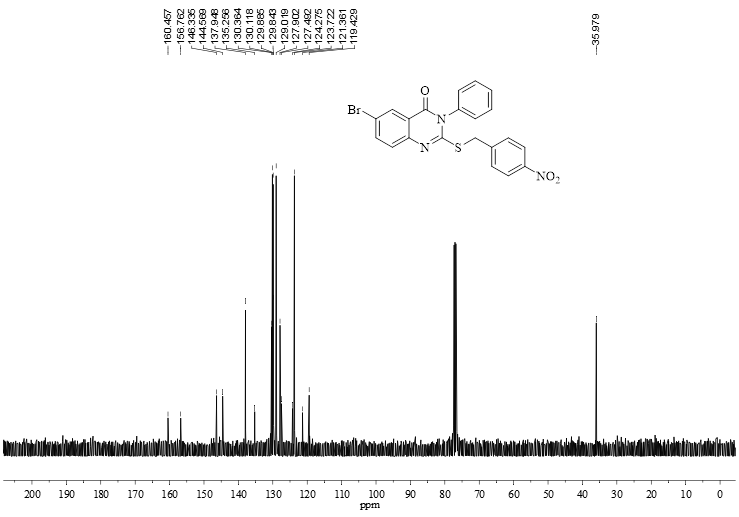


**Figure S23.** ^13^C-NMR spectrum of compound **8h.**


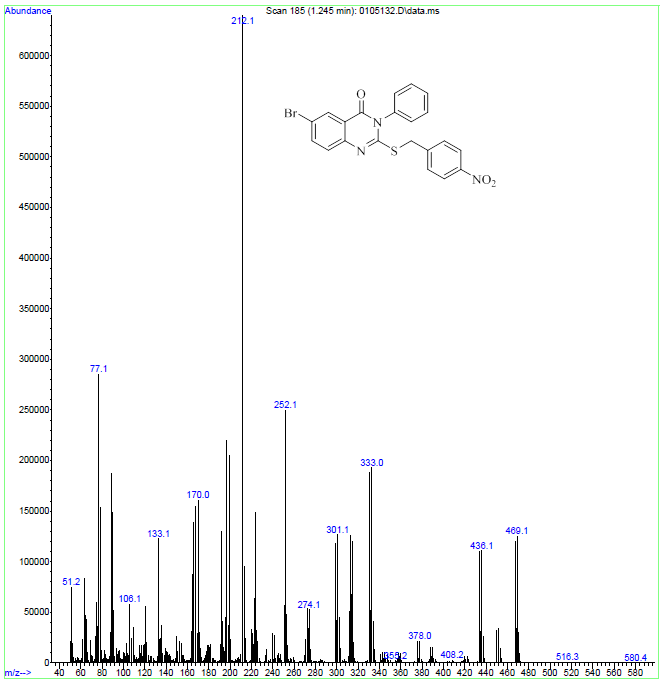


**Figure S24.** Mass spectrum of compound **8h.**

**Table S1.** The calculated total energy (E_tot_), HOMO, LUMO, hardness (ɳ), softness (σ and electron affinity (A) of 8a and 8c

| **Functional** | **Entry** | **E_tot_^a^** | **HOMO^b^** | **LUMO^b^** | **ɳ^b^** | **σ^c^** | **A^b^** |
| --- | --- | --- | --- | --- | --- | --- | --- |
| **B3LYP** | 8a | -3946.24 | -6.12 | -1.41 | 2.35 | 0.21 | 1.41 |
|  | 8c | -3833.11 | -6.14 | -1.57 | 2.28 | 0.22 | 1.57 |
| **CAM-B3LYP** | 8a | -3946.27 | -8.15 | -0.537 | 3.802 | 0.131 | 0.537 |
|  | 8c | -3833.15 | -8.27 | -0.689 | 3.790 | 0.132 | 0.689 |

^a^ in Hartree/particle. ^b^ in ev. ^c^ in ev^-1^

1. *Corresponding authors:

   Soghra Khabnadideh, Tel: +98 -71-32424127-8; Fax: +98-71-32424126; E-mail: khabns@sums.ac.ir

   Sara Sadeghian, Tel: +98 -71-32424127-8; Fax: +98-71-32424126; E-mail: s_sadeghian@sums.ac.ir [↑](#footnote-ref-1)
